# Association Between 24‐h Movement Behaviors and Mental Health in Children and Adolescents: A Systematic Review and Compositional Data Meta‐Analysis

**DOI:** 10.1111/sms.70120

**Published:** 2025-08-19

**Authors:** Matthew Bourke, Hiu Fei Wendy Wang, Kathryn Fortnum, George Thomas, Martin O'Flaherty, Samantha K. Mulcahy, Sjaan R. Gomersall, Tahlia Alsop, Stewart G. Trost, Brianne A. Bruijns, Sophie M. Phillips, Leigh Vanderloo, Patricia Tucker, Kylie D. Hesketh, Matthew Y. W. Kwan, John Cairney

**Affiliations:** ^1^ Health and Wellbeing Centre for Research Innovation, School of Human Movement and Nutrition Sciences The University of Queensland Brisbane Queensland Australia; ^2^ Australian Research Council Centre of Excellence for the Digital Child Kelvin Grove Queensland Australia; ^3^ School of Health and Rehabilitation Sciences The University of Queensland Brisbane Queensland Australia; ^4^ School of Human Movement and Nutrition Sciences The University of Queensland Brisbane Queensland Australia; ^5^ School of Occupational Therapy, Faculty of Health Sciences Western University London Ontario Canada; ^6^ ParticipACTION Toronto Ontario Canada; ^7^ Children's Health Research Institute London Ontario Canada; ^8^ Institute for Physical Activity and Nutrition Deakin University Geelong Victoria Australia; ^9^ Department of Child and Youth Studies, INfant Child and Health Lab Brock University St. Catherines Ontario Canada; ^10^ Department of Family Medicine McMaster University Hamilton Ontario Canada

**Keywords:** cognitive, physical activity, sedentary, sleep, social‐emotional

## Abstract

This systematic review and meta‐analysis synthesized evidence of the association between 24‐h movement behaviors and social–emotional health and cognitive development in children and adolescents aged 3–18 years from studies using compositional data analysis. Systematic literature searches were conducted on five electronic databases from January 2015 to December 2024. Studies were eligible if they assessed sleep, moderate‐to‐vigorous intensity physical activity, light intensity physical activity, and sedentary time. Eligible studies also examined associations between movement behaviors and social–emotional health or cognitive development using compositional data analysis in children and adolescents aged 3–18 years. Pooled effect sizes were estimated using cluster robust variance meta‐analysis. A total of 19 studies (5 longitudinal, 14 cross‐sectional) encompassing 11 826 participants were included. Meta‐analysis showed that engaging in more moderate‐to‐vigorous intensity physical activity, getting more sleep, and spending less time sedentary relative to other movement behaviors was favorably associated with social–emotional health; albeit, the observed relationships were small in magnitude. The association for sleep was stronger among adolescents compared to children. No components of 24‐h movement behavior compositions were associated with cognitive development. These findings suggest that engaging in healthy movement behavior compositions may be beneficial for social–emotional health in young people, although the link between 24‐h movement behavior compositions and cognitive development is less clear.

## 1 Introduction

In 2017, the Canadian 24‐h movement guidelines for children and youth [1] and for the early years [2] were introduced. The first of their kind, these guidelines represented a paradigm shift from considering individual movement behaviors (i.e., sleep, sedentary time, and physical activity) in isolation to viewing them as integrated parts of a whole day [3]. The Canadian 24‐Hour Movement Guidelines were developed based on the best available evidence, including multiple comprehensive systematic reviews [4, 5, 6, 7, 8, 9]. However, when the guidelines were developed, evidence examining the combined effects of 24‐h movement behaviors on child and youth health was limited [10, 11]. Therefore, guideline development remained largely based on evidence of the independent associations of each movement behavior on health outcomes and expert consultations. However, it is important to recognize that these behaviors are interdependent, and spending more time in one behavior will necessitate corresponding decreases in other behaviors [12]. For example, if a child sleeps two more hours a night on average than another child, they will have two fewer hours each day to spend engaging in the remaining behaviors (i.e., sedentary time or physical activity). Therefore, the health benefits or consequences of engaging in one movement behavior result, in part, from not spending that time engaged in an alternative movement behavior [13]. Only considering the independent association between individual movement behaviors and health outcomes, therefore, cannot provide a true representation of what the optimal combinations of 24‐h movement behaviors look like to promote health in children and adolescents.

Alongside the development of the 24‐h movement behavior guidelines, there has been an emerging trend towards the use of compositional data analysis to examine the association between 24‐h movement behaviors and health indicators [14]. Compositional data analysis allows for the consideration of the association between all 24‐h movement behaviors in a single model [15]. It differs from similar methods, including linear isotemporal substitution modeling [16], which analyzes movement behavior data in its raw form (e.g., min/day). In comparison, compositional data analysis expresses movement behaviors as ratios (e.g., time spent sedentary relative to time spent sleeping and being physically active) using log‐ratio transformations [15]. This approach accounts for the co‐dependence of movement behaviors and enables examination of each behavior's relative impact on health outcomes [15].

Mental health outcomes play a vital role in shaping the developmental trajectories and long‐term wellbeing of children and adolescents. The prevalence of many mental health disorders, including internalizing disorders (e.g., depression, anxiety) and externalizing disorders (e.g., conduct disorder, attention‐deficit‐hyperactivity disorder), increases dramatically during childhood and reaches its peak during adolescence and young adulthood [17, 18]. Importantly, mental health extends beyond the mere absence of illness. The World Health Organization defines mental health as a state of wellbeing in which an individual can realize their abilities [19]. In children, a state of wellbeing includes having a positive sense of identity, the ability to manage thoughts and emotions, being able to build and sustain relationships with others, and the aptitude to learn and acquire an education [20]. Therefore, mental health encompasses both social–emotional health (e.g., internalizing and externalizing symptoms, resilience, prosocial behaviors) and cognitive development (e.g., self‐regulation, executive functioning, memory) components. Although mental health can be broken into its constituent parts such as emotional problems, social functioning, wellbeing, and mental illness, the World Health Organization's definition exemplifies a complete state approach recognizing that overall social–emotional health represents a key indicator [21].

The independent associations of physical activity [22, 23], sedentary behavior [22, 24, 25], and sleep [26, 27, 28] with social–emotional health and cognitive development in children and adolescents have been examined in multiple systematic reviews and meta‐analyses. These reviews show that, generally speaking, spending more time active (especially in moderate‐to‐vigorous physical activity [MVPA]), less time sedentary (especially sedentary screen time), and more time sleeping is related to more favorable mental health outcomes. However, less is currently known about the association between 24‐h movement behavior compositions and social–emotional and cognitive outcomes in children. Recent attempts to synthesize the existing evidence have been limited by the small number of studies examining associations between 24‐h movement behavior compositions and mental health outcomes, restrictive inclusion criteria limiting the scope of the review, and absence of quantitative synthesis of the results [29, 30, 31]. Findings from recent reviews have been mixed. For example, Dale, O'Rourke, Nussbaumer‐Streit, Probst [32] found consistent evidence that spending more time sedentary compared to other movement behaviors was related to worse social–emotional outcomes in children and adolescents, whereas another systematic review found that the vast majority of research using compositional data analysis has found a null association [33]. Considering cognitive outcomes, Kuzik, Duncan, Beshara, MacDonald, Silva, and Tremblay [33] found consistent evidence that spending more time in light intensity physical activity relative to other behaviors was related to worse cognitive outcomes in children. Given the exponential growth in published research on 24‐h movement behaviors in recent years [34], a quantitative synthesis of the association between 24‐h movement behaviors and mental health in children and adolescents is warranted. Such findings could help identify optimal combinations of 24‐h movement behaviors to support social–emotional and cognitive development in children and adolescents. Therefore, the aim of this study was to systematically identify and quantitatively synthesize published research using compositional data analysis to examine the association between 24‐h movement behaviors and mental health in children and youth.

## 2 Methods

This systematic review and meta‐analysis was registered with the International Prospective Register of Systematic Reviews database (CRD42024502473) and conducted and reported in accordance with the Preferred Reporting Items for Systematic Reviews and Meta‐Analyses [35].

### 2.1 Information Sources and Literature Search

Initially, a systematic literature search was conducted in Medline (via OVID), PsychInfo, SPORTDiscus, EMBASE, and SCOPUS from 2015 (when the first compositional data analysis paper of 24‐h movement behaviors was published) to January 11, 2024. The literature search was updated on December 5, 2024. The search was conducted using key terms for 24‐h movement behaviors, mental health, and children and youth. A full list of search terms can be seen in Appendix A1 (Table A1). Primary literature searches were supplemented by manual searches to identify any potentially relevant articles not identified in the primary literature search (e.g., articles published ahead of print which were yet to be indexed) in the following journals: European Journal of Sports Science; Applied Physiology, Nutrition and Metabolism; Plos One; International Journal of Environmental Research and Public Health; Journal of Activity, Sedentary and Sleep Behaviors; Journal of Sport Sciences; Journal of Physical Activity and Health; International Journal of Behavioral Nutrition and Physical Activity; and BMC Public Health. These journals were selected as they appeared most regularly among studies included in the full‐text screening and were therefore considered the most likely to publish manuscripts relevant to this review. Additionally, the reference lists of all included studies were manually screened to supplement the primary literature search.

### 2.2 Eligibility Criteria

#### 2.2.1 Participants

Studies were eligible for inclusion in this review if they reported on children and adolescents between the ages of 3 and 18 years. Children under 3 were excluded from the review because 24‐h movement behavior guidelines for children younger than 3 do not include specific recommendations for MVPA [2, 36]. Although some definitions of adolescents include people aged older than 18 [37], 24‐h movement guidelines are different for people aged over 18 years [38], so the upper limit was set at 18 years. No exclusion criteria were placed on children who were within the eligible age range.

#### 2.2.2 Study Design

All observational studies (e.g., cross‐sectional studies, longitudinal cohort studies) were eligible for inclusion in this review. Intervention studies were eligible if they reported on associations between 24‐h movement behaviors and social–emotional health or cognitive development at baseline before any intervention was administered, or at follow‐up while controlling for intervention effects.

#### 2.2.3 Outcome Measures

Studies which reported on any aspect of social–emotional health (e.g., internalizing symptoms, externalizing behaviors, prosocial behaviors, wellbeing) or cognitive development (e.g., self‐regulation, academic achievement, executive functioning) were included in the review.

#### 2.2.4 Exposure

Only studies which reported on all components of 24‐h movement behaviors (i.e., sleep, sedentary time, light intensity physical activity [LPA], MVPA) using compositional data analysis were included in this review. Studies which only reported on a subset of 24‐h movement behaviors (e.g., did not include sleep and only reported on wake time movement behaviors) and/or did not use compositional data analysis were excluded.

### 2.3 Study Selection

The screening of titles and abstracts identified in the initial literature search was aided through the use of ASReview, an open‐source machine learning program developed to expedite the review process [39]. ASReview uses text contained within each title and abstract to train a machine learning model which is used to rank titles and abstracts based on the expected relevance to the review. Reviewers select if the title and abstract presented to them is relevant, and the machine learning model is continuously trained based on the reviewer's selection. In reviews with a large number of studies (i.e., > 5000), this process is often able to identify 100% of relevant records after screening 30% of all titles and abstracts [39]. Similar to previous studies [40], two independent reviewers screened titles and abstracts until at least 30% of all titles and abstracts had been screened and they had labeled 500 consecutive titles and abstracts as irrelevant. In screening at least 30% of titles and abstracts, reviewers are likely to make different decisions on some records (i.e., one reviewer may decide a record is relevant while the other decides it is irrelevant, as is standard for all title and abstract screening processes). The result will be a different machine learning model for each reviewer, ultimately meaning a slightly different pool of titles and abstracts will be screened by each. All titles and abstracts identified in the updated literature search (i.e., between January 2024 and December 2024) were screened by both reviewers in Covidence (Veritas Health Innovation, Melbourne, Australia, https://www.covidence.org). All titles and abstracts that were labeled as relevant by at least one reviewer were retrieved for full‐text screening which was conducted using Covidence. Each full text was reviewed by two independent reviewers. If there was a discrepancy between the two independent reviewers' decisions on a full text (proportion disagreement = 2.6%), the lead author (who was one of the original independent reviewers) considered the reason that the other reviewer disagreed with their decisions, re‐read the full text, and made the final decision on whether the full text was included.

### 2.4 Data Extraction

Study characteristics were extracted by a single reviewer and cross‐checked for all included studies by a second author. Extracted data on study characteristics included the country in which the study was conducted, the number of participants and their sex and age, how 24‐h movement behaviors were assessed, the tools used to measure social–emotional health and cognitive development, and the study design. Compositional data analysis uses log‐ratio transformations to model the association between all 24‐h movement behaviors in a single model [15]. Therefore, the regression coefficient for the first log‐ratio coordinate for each behavior was extracted to calculate the effect size for the meta‐analyses by a reviewer with expertise in compositional data analysis. If a manuscript did not report the regression coefficient for the first log‐ratio coordinate for each behavior in text, it was manually estimated by the lead author using the original data from the included study (*k* = 2). If the data were not publicly available, they were requested from the corresponding author (*k* = 2).

### 2.5 Quality Assessment

The quality of included studies was examined using the Appraisal tool for Cross‐Sectional Studies [AXIS] [41]. AXIS is a 20‐item quality appraisal tool which assesses the quality of reporting and study design. Three additional items were assessed for longitudinal studies including whether mental health at baseline was controlled for in the longitudinal model, whether the follow‐up length was long enough to see a longitudinal association, and whether missing longitudinal data was related to exposure or outcome variables. The quality of each study was assessed by two independent reviewers. Where there were discrepancies in quality rating among reviewers, the reviewers met to discuss the study quality to reach consensus. Overall quality was reported as the percentage of applicable quality criteria that a paper received a favorable appraisal on.

### 2.6 Calculation of Effect Sizes

Effect size was calculated as the standardized absolute change in social–emotional health or cognitive development outcomes for a relative change in movement behavior composition. The approach used to calculate the effect sizes has been published previously [15]. Specifically, the effect size was calculated as:

$$\frac{\beta_{\mathrm{ilr}1}\times \sqrt{\frac{3}{4}}\times \ln\left(\frac{1+r}{1-s}\right)}{{\mathrm{SD}}_{y}}$$

where $\beta_{\mathrm{ilr}1}$ is the estimated regression coefficient for the first log‐ratio coordinate, r is the percentage increase in the first compositional part, s is the corresponding percentage decrease in the remaining compositional parts and ${\mathrm{SD}}_{y}$ is the standard deviation of the outcome. The effect size was expressed as changes in standard deviations to account for the variability in scales used to assess outcomes. All effect sizes were scored so that positive effects were favorable (i.e., better mental health). Two studies reported sedentary screen time and non‐screen sedentary behaviors separately [42, 43]. The effect sizes for sedentary screen time and non‐screen sedentary time were aggregated into a single effect size to be included in the meta‐analyses. The standard error of the effect size was calculated as:

$$\frac{\sqrt{\frac{3}{4}}\times \ln\left(\frac{1+r}{1-s}\right)\times \sqrt{\mathrm{var}\left(\beta_{\mathrm{ilr}1}\right)}}{{\mathrm{SD}}_{y}}$$

Standardized absolute changes in outcomes for relative changes in movement behavior compositions were estimated in 10‐min increments up to 60 min/day. One study reported on log‐transformed outcomes [44]. The regression coefficient from this study was transformed to absolute values to facilitate its inclusion in the meta‐analysis [45].

### 2.7 Meta‐Analyses

All analyses were conducted using R v. 4.1.3 (R Core Team, Vienna, Austria) and R studio v. 1.3 (RStudio Team, Boston, MA). Pooled effects were estimated using random‐effects meta‐analysis with a robust variance estimator to account for the inclusion of correlated effect sizes within included studies [46] using the metafor [47] and clubSandwich [48] packages. Specifically, the correlated and hierarchical effects model [49] was used to estimate the pooled effects. The correlated and hierarchical effects model can model complex dependency structures, such as between repeated assessments in longitudinal studies and between multiple outcome assessments within a single study, by modeling both between‐study and within‐study heterogeneity in effect sizes and accounting for a correlation between multiple effect sizes from the same study. The model was estimated with an assumed correlation of *ρ* = 0.6 between effect sizes within studies. Cluster robust standard errors were estimated using a sandwich estimator with a small sample adjustment [50]. Effect sizes were weighted based on the inverse of their variance. Variance between studies (*𝜏* ^2^) and between effect sizes within‐study (*ω* ^2^) were estimated using the restricted maximum likelihood method, and the percentage of total heterogeneity explained at each level of analysis was calculated (*I* ^2^) using the dmetar [51] package. Separate meta‐analyses were performed for social–emotional health and cognitive development. Results were plotted using the ggplot2 [52] and ggpubr [53] packages.

Publication bias was assessed by examining funnel plot asymmetry. Funnel plot asymmetry was assessed using the “Egger sandwich test” [54]. Essentially, this test is a meta‐regression where effect sizes are regressed on a measure of their precision (i.e., standard error). A significant association (*p* < 0.05) between an effect size and their standard errors indicates publication bias. A small sample adjustment was applied to the Satterthwaite degrees of freedom for the Egger sandwich test to reduce the Type I error [50].

Within‐study and between‐study subgroup analyses were conducted to determine effect modifiers of the association between each component of the 24‐h movement behavior composition and social–emotional and cognitive outcomes. Within‐group subgroup analysis was conducted to determine if the association between each part of the 24‐h movement behavior differed based on different social–emotional (i.e., internalizing, externalizing, prosocial, positive mental health) and cognitive development (i.e., inhibition, memory) outcomes. Within‐study subgroup analysis was conducted using a subgroup correlated effects model [49]. Between‐study subgroup analysis was conducted to determine if the association between each part of the 24‐h movement behavior with social–emotional health differed based on participants' age (i.e., < 10 years, ≥ 10 years). Because subgroup analyses with very small degrees of freedom may be unreliable [50], subgroup analyses were guided by data availability and was only conducted for subgroups that consisted of at least five studies. Therefore, studies examining young children (3–4 years) and older children (5–9 years) were combined and compared to studies on adolescents (10–18 years).

## 3 Results

### 3.1 Study Selection

**FIGURE 1:**
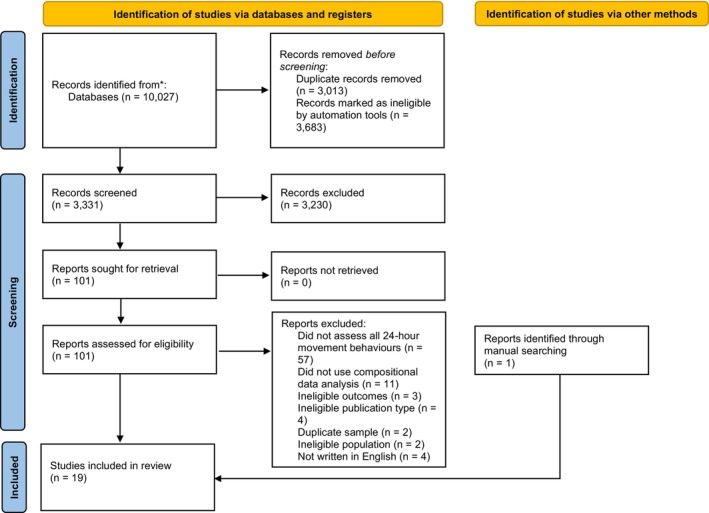
PRISMA flowchart.

A PRISMA diagram of the study selection process is in Figure 1. After removing duplicates, the initial study search identified 5785 unique papers and the updated search identified 1229 additional studies. Of these, 3331 titles and abstracts were manually screened. Each reviewer had a unique machine learning model based on their individual choices; therefore, 2802 titles and abstracts were screened by both reviewers and 529 were screened by a single reviewer. The remaining 3683 titles and abstracts were ranked below the threshold to warrant manual screening by both reviewers' machine learning algorithm and were automatically excluded. A total of 101 titles and abstracts were selected as potentially relevant by at least one reviewer and sought for full‐text review. Of these, 83 articles were excluded (see Figure 1) and 18 full texts were included. One additional article was identified through supplementary searches, resulting in 19 studies included in the meta‐analyses.

### 3.2 Study Characteristics

A detailed description of study characteristics is displayed in Table 1. Most studies were conducted with adolescents (10–18 years; *k* = 7) or young children (3–4 years; *k* = 7), and children (5–9 years; *k* = 4). One study included a sample of children and adolescents (6–17 years). All participants were children recruited from the general population, except for those in one study [63] which were ‘left‐behind’ children (i.e., children nurtured by a caregiver who is not their parent). Four studies were conducted in Australia [55, 57, 64, 69] and China [43, 61, 62, 63], three in Canada [42, 44, 60], two in Brazil [56, 58] and Singapore [65, 67], and one each in the United Kingdom [59], New Zealand [68], and the United States [66]. Additionally, one study reported on multiple samples from multiple countries [70]. The sample size of included studies ranged from 95 to 4169 participants (median = 351.5). All studies used accelerometers to assess sedentary time, LPA, and MVPA, while 13 studies used accelerometers to assess sleep duration and six used parent‐reported sleep duration. Slightly more studies used hip‐worn accelerometers (*k* = 11) compared to wrist‐worn accelerometers (*k* = 8). All studies used cut‐points of either raw acceleration data or proprietary accelerometer counts per epoch. A total of 12 different accelerometer cut‐points were used across the included studies to estimate physical activity and sedentary time. Two studies also assessed sedentary screen time using parent‐reported questionnaires [42, 63]. Each of these studies also assessed overall sedentary time using accelerometers. Social–emotional outcomes reported included internalizing symptoms (*k* = 10), externalizing symptoms (*k* = 8), social functioning (*k* = 7), positive mental health (e.g., self‐esteem, emotional wellbeing; *k* = 3), overall psychosocial difficulties (*k* = 2), and emotional self‐control (*k* = 2). Cognitive outcomes reported included memory (*k* = 7), inhibition (*k* = 7), cognitive flexibility (*k* = 4), academic achievement (*k* = 3), executive functioning (*k* = 3), vocabulary (*k* = 3), attention (*k* = 2), cognitive self‐regulation (*k* = 1), behavioral self‐regulation (*k* = 1), effortful control (*k* = 1), and hyperactivity (*k* = 1). The measures used to assess each of the outcomes are detailed in Table 1. Five studies employed a longitudinal study design, while the remaining 14 studies were cross‐sectional in nature.

**TABLE 1:** Description of study characteristics.

Study	Country design	Participants, age, and gender/sex	24‐h movement behavior measurement	Outcome measurement	Covariates included in models
Bourke et al. [55]	Australia Longitudinal (4‐year follow‐up)	*N* = 361 Age = 6 years Sex = 49.6% female	Sedentary, LPA, & MVPA: GENEActive Sleep: Sleep log	**Social–Emotional** Internalizing problems (SDQ) Externalizing problems (SDQ) Prosocial behavior (SDQ)	Sex Ethnicity Mother's education Mother's employment
Bezerra et al. [56]	Brazil Cross‐sectional	*N* = 123 Age = 4.6 years Sex = 50.4% female	Sedentary, LPA, & MVPA: ActiGraph WGT3‐X Sleep: Parent‐reported	**Cognitive** Inhibitory control (EYT) Working memory (EYT) Cognitive flexibility (EYT)	Age BMI Sex
Carson et al. [44]	Canada Cross‐sectional	*N* = 4169 Age = 11.4 years Sex = 48.7% female	Sedentary, LPA, & MVPA: Actical Sleep: Parent/child reported	**Social–Emotional** Overall strengths and difficulties (SDQ)	Age Sex Highest household education
Chong et al. [57]	Australia Longitudinal (1‐year follow‐up)	*N* = 127 Age = 11.7 years Sex = 57.5% female	Sedentary, LPA, MVPA & Sleep: GENEActive	**Social–Emotional** Internalizing problems (SDQ) Externalizing problems (SDQ) Prosocial behavior (SDQ) Psychological distress (K‐10)	Age Sex Socioeconomic status BMI Pubertal progression Primary‐secondary school transition experience
de Faria et al. [58]	Brazil Cross‐sectional	*N* = 217 Age = 16 years Sex = 49.3% female	Sedentary, LPA, MVPA & Sleep: ActiGraph GT3X	**Social–Emotional** Depression/Anxiety (GHQ‐12) Social dysfunction (GHQ‐12)	Age Sex Socioeconomic status BMI Body mass index z‐score
Fairclough et al. [59]	UK Cross‐sectional	*N* = 359 Age = 11.5 years Sex = 50.7% female	Sedentary, LPA, MPA, VPA & Sleep: ActiGraph GT9X	**Cognitive** Switching errors (CANTAB) Spatial working memory (CANTAB) Inhibition (CANTAB) **Social–Emotional** Internalizing problems (SDQ) Externalizing problems (SDQ) Depression (MFQ) Self‐esteem (RSES)	Age Sex Ethnicity Socioeconomic status Indices of Multiple Deprivation BMI
Kuzik et al. [60]	Canada Cross‐sectional	*N* = 95 Age = 4.5 years Sex = 30.5% female	Sedentary, LPA, MVPA & Sleep: ActiGraph wGT3X‐BT	**Cognitive** Inhibitory control (EYT) Working memory (EYT) Language development (EYT) Behavioral self‐regulation (CSBQ) Cognitive self‐regulation (CSBQ) **Social–Emotional** Emotional self‐regulation (CSBQ) Externalizing (CSBQ) Internalizing (CSBQ) Sociability (CSBQ) Prosocial behavior (CSBQ)	Age Sex Ethnicity Hours of childcare attendance Number of siblings Parental age, relation to child, education, income, marital status, home type, yard type
Lau et al. [61]	China Cross‐sectional	*N* = 426 Age = 3.8 years Sex = 45.8% female	Sedentary, LPA, MVPA: ActiGraph GT3‐BT Sleep: Parent‐reported	**Cognitive** Inhibitory control Working memory Cognitive flexibility	Age Sex Height Weight BMI Grade
Li et al. [43]	China Cross‐sectional	*N* = 205 Age = 4.8 years Sex = 42.9% female	Sedentary, LPA, MVPA: wGT3X‐BT Sleep: Parent‐reported Sedentary screen time: Parent‐reported	**Social–Emotional** Internalizing problems (SDQ) Externalizing problems (SDQ) Prosocial behavior (SDQ)	Age Sex BMI Socioeconomic status
Lu et al. [62]	China Cross‐sectional	*N* = 135 Age = 4.55 years Sex = 49.6% male	Sedentary, LPA, MVPA: Actigraph GT9X Sleep = Parent reported	**Cognitive** Inhibitory control Working memory Cognitive flexibility	Age Sex BMI
Lu et al. [63]	China Cross‐sectional	*N* = 275 Age = 12.9 years Sex = 55.6% female	Sedentary, LPA, MVPA & Sleep: ActiGraph GT3X	**Social–Emotional** Internalizing problems (SDQ) Externalizing problems (SDQ) Prosocial behavior (SDQ)	Age Sex BMI Siblings Socioeconomic status Education status Left‐behind status
Ng et al. [64]	Australia Cross‐sectional	*N* = 1181 Age = 12 years Sex = 49% female	Sedentary, LPA, MVPA & Sleep: GENEActive	**Social–emotional** Psychosocial functioning (PedsQL)	Age Sex Socioeconomic position Pubertal status
Padmapriya et al. [65]	Singapore Longitudinal (2.5‐year follow‐up)	*N* = 432 Age = 8 years Sex = 47.2%	Sedentary, LPA, MVPA & Sleep: ActiGraph GT3X+	**Cognitive** Executive functioning (NESPY‐II) Academic achievement (WIAT‐III)	
Roremet al. [42]	Canada Cross‐sectional	*N* = 575 Age = 5 years Sex = 49.6% female	Sedentary, LPA, MVPA & Sleep: ActiGraph GT3X‐BT (NDR; Chandler VM) Sedentary screen time = Parent‐reported	**Social–emotional** Internalizing problems (CBCL) Externalizing problems (CBCL)	Sex Ethnicity Family income Marital status Siblings
St. Laurent et al. [66]	USA Cross‐sectional	*N* = 388 Age = 4.3 years Sex = 44.4% female	Sedentary, LPA, MVPA & Sleep: Actiwatch	**Cognitive** Receptive vocabulary (PPVT‐IV) Visuospatial memory Procedural memory Executive attention **Social–emotional** Internalizing behavior (CBCL) Externalizing behavior (CBCL)	Age Sex Race Ethnicity Socioeconomic status
Tan et al. [67]	Singapore Longitudinal (2‐year follow‐up)	*N* = 370 Age = 8–10 years Sex = 50.5% female	Sedentary, LPA, MVPA & Sleep: ActiGraph wGT3X‐BT	**Social–emotional** Emotional wellbeing (KINDL‐Kid) Self‐esteem (KINDL‐Kid) Relationship with family (KINDL‐Kid) Relationship with friends (KINDL‐Kid) School functioning (KINDL‐Kid)	Age Sex Ethnicity Maternal education Socioeconomic status BMI
Taylor et al. [68]	New Zealand Longitudinal (1.5‐year follow‐up)	*N* = 344 Age = 5 years Sex = 49.7% female	Sedentary, LPA, MVPA & Sleep: Actical	**Cognitive** Inhibitory control (NEPSY‐2) Hyperactivity (BASC‐2) Executive functioning (BASC‐2) Attentional problems (BASC‐2) **Social–emotional** Anxiety (BASC‐2) Depression (BASC‐2) Resilience (BASC‐2) Emotional self‐control (BASC‐2)	Maternal age at child's birth Sex Weight Height BMI z‐score
Watson et al. [69]	Australia Cross‐sectional	**ISCOLE** *N* = 294 Age = 10.7 years Sex = 54.8% females **LSAC** *N* = 940 Age = 12.0 years Sex = 47.8% females	Sedentary, LPA, MVPA & Sleep = Actigraph GT3X+	**Cognitive** Academic achievement (NAPLAN)	Age Sex Socioeconomic position Highest parental education Pubertal status
Zahran et al. [70]	Australia, Norway, Canada Cross‐sectional	*N* = 858 Age = 4.2 years Sex = 44.9% female	Sedentary, LPA, MVPA & Sleep = Actigraph GT3X+	**Cognitive** Inhibitory control (EYT) Working memory (EYT) Vocabulary (EYT)	Age Sex Parental education Marital status Study

Abbreviations: BASC‐2, Behavioral Assessment System for Children; CANTAB, CANTAB Connect (Cambridge Cognition Ltd); CBCL, Child Behavior Checklist; CSBQ, Child Self‐Regulation and Behavior Questionnaire; EYT, Early Years Toolbox; GHQ, General Health Questionnaire; ISCOLE, The International Study of Childhood Obesity, Lifestyle and the Environment; K10, Kessler 10; LSAC, Longitudinal Study of Australian Children; MFQ, Mood and Feelings Questionnaire; NAPLAN, National Assessment Program—Literacy and Numeracy; PPVT‐IV, Peabody Picture Vocabulary Test 4th Edition; RSES, Rosenberg Self‐Esteem Scale; SDQ, Strengths and Difficulties Questionnaire; WIAT‐III, Wechsler Individual Achievement Test, Third Edition.

### 3.3 Quality Appraisal

The full results of the study quality assessment are reported in Appendix B1 (Table B1). Overall study quality ranged from 70% to 90%. All studies had clear aims, used an appropriate study design, appropriate assessments, and clear methods. Ten studies employed a representative selection process; 7 studies reported on variables related to non‐response, and 2 studies provided a rationale for the sample size. Among the longitudinal studies, all had sufficient time between baseline and follow‐up to observe a longitudinal association; 4 of 5 studies demonstrated that missing longitudinal data was not related to outcome or exposure variables, while only 1 study controlled for baseline mental health in longitudinal models.

### 3.4 Meta‐Analyses

#### 3.4.1 Social–Emotional Health

**FIGURE 2:**
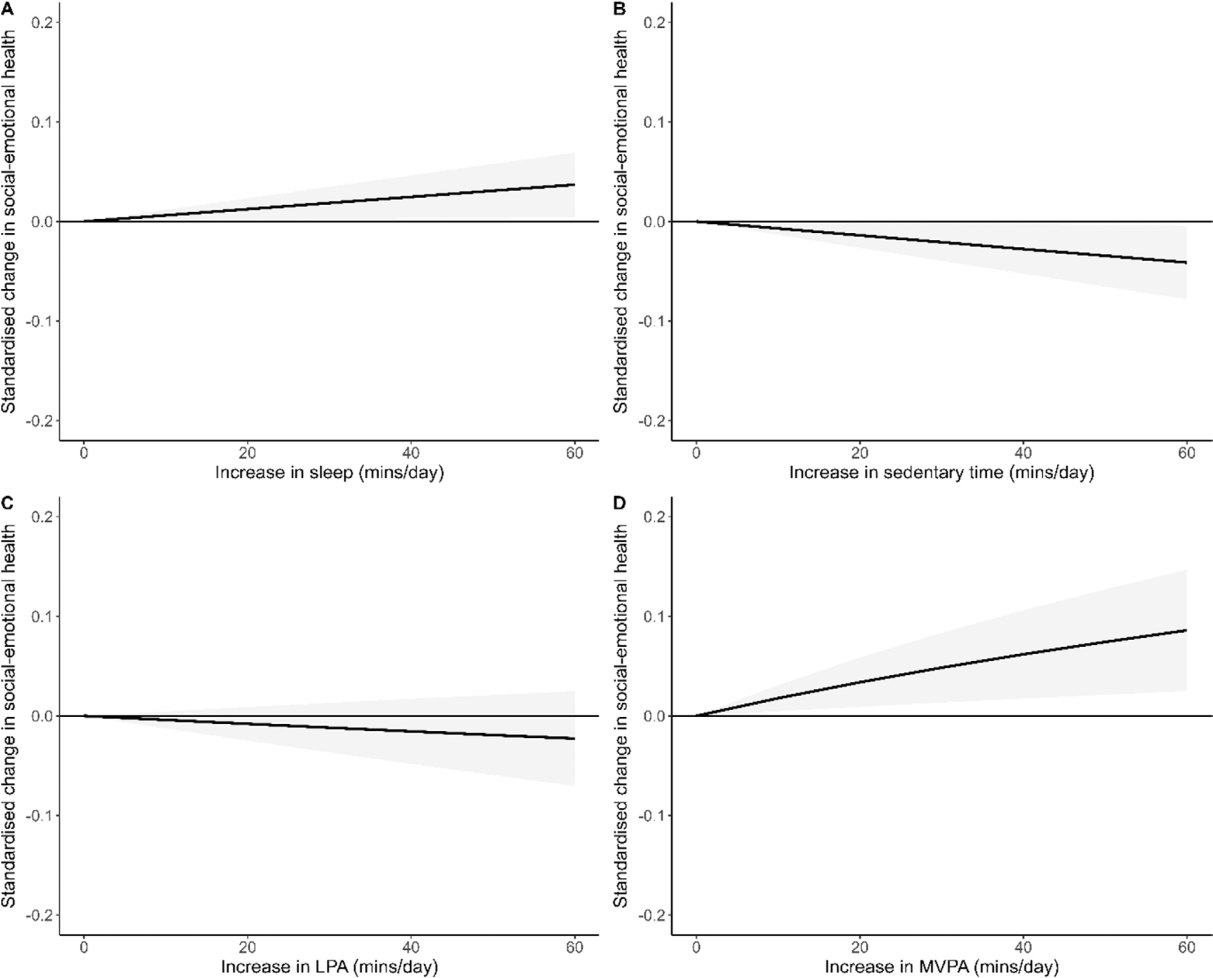
Association between relative time spent (A) sleeping, (B) sedentary, (C) engaged in LPA, and (D) engaged in MVPA, and social–emotional health.

Overall, 14 studies (*n* = 8951 participants) reporting 58 effect sizes were included in the meta‐analyses for social–emotional health. Results are presented in Figure 2 and individual effect sizes are in Appendix C1 (Figures C1–C4). Spending more time in MVPA, *t*(7.94) = 3.10, *p* = 0.015, *I* ^2^_(level2)_ = 81.0%, *I* ^2^_(level3)_ = 0.0%, relative to other movement behaviors, was positively associated with social–emotional health. Spending more time sleeping, *t*(9.20) = 2.59, *p* = 0.029; *I* ^2^_(level2)_ = 52.4%, *I* ^2^_(level3)_ = 0.0%, relative to other movement behaviors, was also positively associated with social–emotional health. Spending more time in sedentary behavior relative to other behaviors was negatively associated with social–emotional health, *t*(9.94) = −2.49, *p* = 0.032, *I* ^2^_(level 2)_ = 31.7%, *I* ^2^_(level 3)_ = 39.6%. The amount of time spent in LPA relative to other movement behaviors, while in the same direction as sedentary behavior, was not significantly associated with social emotional health, *t*(7.6) = −1.10, *p* = 0.304, *I* ^2^_(level2)_ = 71.8%, *I* ^2^_(level3)_ = 0.0%. Funnel plots for social–emotional health are displayed in Appendix D1 (Figures D1–D4). The Egger's test did not show evidence of publication bias (*p* > 0.05).

Results from the subgroup analysis for social emotional outcomes are displayed in Table 2. There were no significant differences in the association between any component of 24‐h movement behavior composition and various social–emotional health outcomes. Nevertheless, the results did show that time spent in MVPA had a favorable association with internalizing symptoms, social functioning, and positive mental health, but was not related to better externalizing behaviors. Participants' age did significantly moderate the association between sleep and social–emotional health. Time spent sleeping relative to other movement behaviors was related to better social–emotional health among adolescents but was not related to social–emotional health among children. There were no significant differences in the association between sedentary time, LPA, or MVPA with social–emotional health based on participants' age.

**TABLE 2:** Subgroup analysis for social–emotional health.

Movement behavior|Subgroup	df	Effect size	95% confidence interval	Subgroup *p*
Sleep				
Outcome^a^				
Externalizing	3.81	0.028	−0.009, 0.064	0.796
Internalizing	5.37	0.021	−0.009, 0.051	
Social functioning	3.16	0.024	−0.008, 0.055	
Positive mental health	1.68	0.013	−0.019, 0.045	
Age				
< 10 years	4.27	−0.007	−0.014, 0.013	0.017
≥ 10 years	4.25	0.033	0.005, 0.060	
Sedentary				
Outcome^a^				
Externalizing	2.98	−0.011	−0.031, 0.009	0.412
Internalizing	7.24	−0.024	−0.049, 0.002	
Social functioning	5.08	−0.024	−0.070, 0.002	
Positive mental health	3.38	−0.009	−0.045, 0.027	
Age				
< 10 years	4.94	−0.035	−0.078, 0.009	0.240
≥ 10 years	4.39	−0.011	−0.035, 0.014	
LPA				
Outcome^a^				
Externalizing	5.88	0.019	−0.061, 0.098	0.636
Internalizing	7.20	0.002	−0.059, 0.064	
Social functioning	5.17	−0.039	−0.120, 0.042	
Positive mental health	1.29	−0.021	−0.043, 0.001	
Age				
< 10 years	3.50	−0.003	−0.041, 0.034	0.257
≥ 10 years	4.64	−0.027	−0.067, 0.012	
MVPA				
Outcome^a^				
Externalizing	3.46	−0.019	−0.069, 0.032	0.195
Internalizing^a^	6.82	0.062	−0.012, 0.135	
Social functioning	4.10	0.088	−0.019, 0.194	
Positive mental health	3.85	0.094	−0.027, 0.216	
Age				
< 10 years	9.36	0.056	0.011, 0.101	0.480
≥ 10 years	3.54	0.030	−0.046, 0.1	

^a^ All outcomes were scored, so a positive association is favorable (i.e., better social emotional health).

#### 3.4.2 Cognitive Development

**FIGURE 3:**
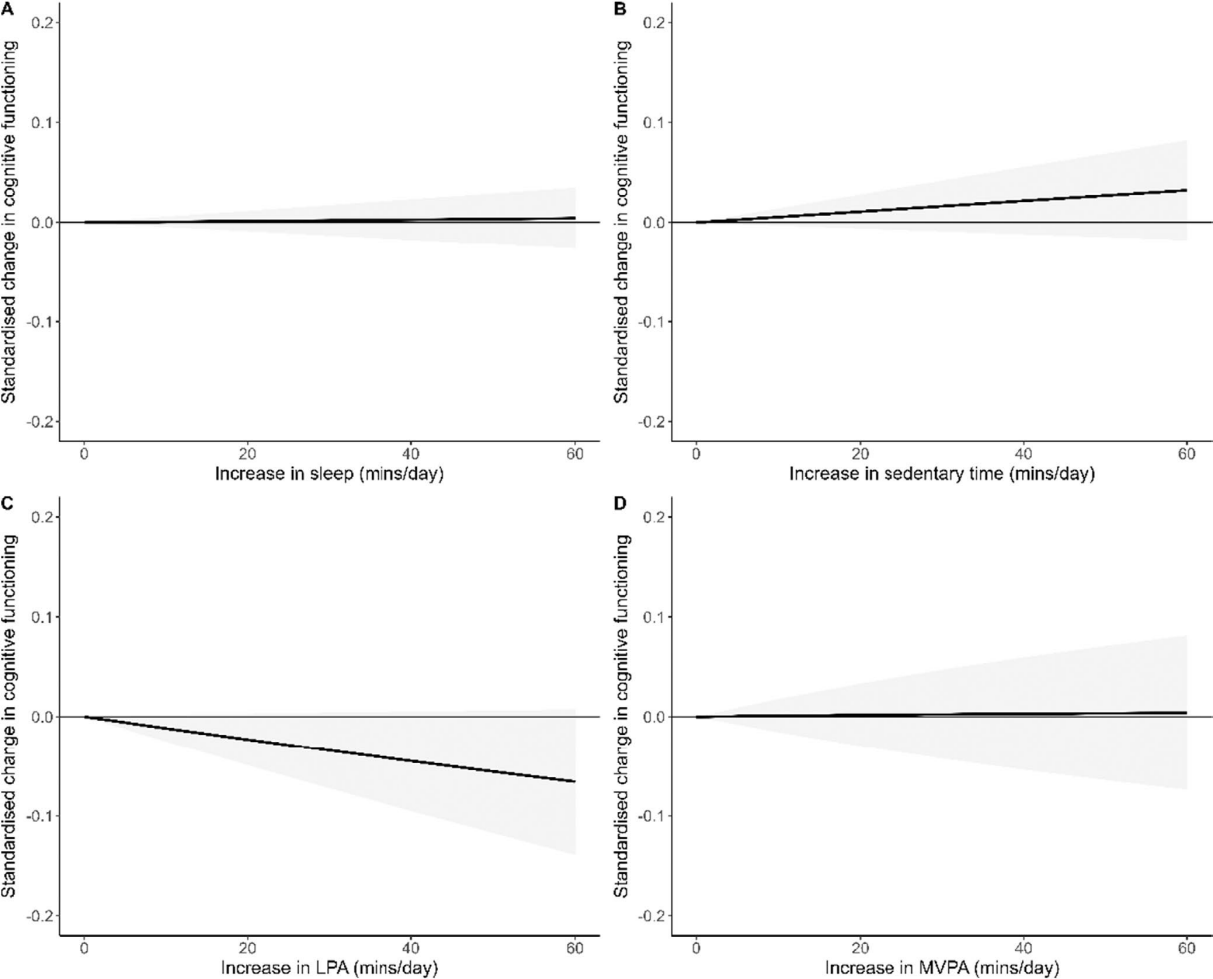
Association between relative time spent (A) sleeping, (B) sedentary, (C) engaged in LPA, and (D) engaged in MVPA, and cognitive development.

A total of 10 studies reporting on 11 unique samples (*n* = 4303 participants) and 41 effect sizes were included in the meta‐analysis for cognitive development. Results are displayed in Figure 3 and individual effect sizes are in Appendix C1 (Figures C5–C8). The amount of time spent sleeping, *t*(6.95) = 0.32, *p* = 0.760, *I* ^2^_(level 2)_ = 57.0%, *I* ^2^_(level 3)_ = 0.0%; sedentary, *t*(8.87) = 1.46, *p* = 0.180, *I* ^2^_(level 2)_ = 34.4%, *I* ^2^_(level 3)_ = 33.7%; engaged in LPA, *t*(7.58) = −2.08, *p* = 0.073, *I* ^2^_(level 2)_ = 76.1%, *I* ^2^_(level 3)_ = 1.2%; or engaged in MVPA, *t*(7.17) = 0.13, *p* = 0.903, *I* ^2^_(level 2)_ = 74.4%, *I* ^2^_(level 3)_ = 0.0% relative to other movement behaviors were not significantly related to cognitive development in children and adolescents. Funnel plots for cognitive development are displayed in Appendix D1 (Figures D5–D8). The Egger's test found no significant evidence of publication bias (*p* > 0.05).

Results for the subgroup analysis for cognitive development outcomes are presented in Table 3. Although there were no significant differences in the association between components of the 24‐h movement behavior compositions with inhibition and memory, there was a clear trend that the association may be stronger for inhibition.

**TABLE 3:** Subgroup analysis of difference in association between 24‐h movement behavior compositions and different cognitive outcomes.

Outcome	df	Effect size	95% Confidence Interval	Subgroup *p*
Sleep				
Inhibition	3.19	−0.023	−0.076, 0.030	0.366
Memory	2.98	0.001	−0.044, 0.046	
Sedentary				
Inhibition	3.77	0.037	−0.027, 0.101	0.510
Memory	2.87	0.023	−0.007, 0.053	
LPA				
Inhibition	2.51	−0.106	−0.198, −0.014	0.206
Memory	4.57	−0.029	−0.159, 0.101	
MVPA				
Inhibition	1.84	0.058	−0.042, 0.158	0.272
Memory	2.46	0.021	−0.064, 0.105	

*Note:* All outcomes were scored, so a positive association is favorable (i.e., better cognitive development).

## 4 Discussion

The aim of this systematic review and meta‐analysis was to synthesize research using compositional data analysis to examine associations between 24‐h movement behaviors and social–emotional health and cognitive development in children and adolescents. Results demonstrated that time spent in MVPA and sleeping, relative to other behaviors, was positively associated with social–emotional health; whereas time spent sedentary relative to other behaviors was negatively associated with social–emotional health. No components of 24‐h movement behavior compositions were significantly related to cognitive development.

The results from the current review suggest that decreasing the amount of time spent sedentary and replacing it with either sleep or MVPA may promote better mental health outcomes in children and adolescents, thus providing further support for the 24‐h movement guidelines. These results build on evidence that has established the independent associations between physical activity [71, 72], sedentary time [24, 73], and sleep [26, 74], with social–emotional health in children and adolescents. Similar results have been reported in studies examining the association between meeting all the 24‐h movement guideline and mental health outcomes in young people [31]. However, it is important to note that, despite being statistically significant, the magnitude of the association between components of 24‐h movement behavior composition and social–emotional health was small. For example, engaging in 60 more minutes of MVPA while simultaneously decreasing the amount of time spent in the remaining movement behaviors was only associated with a 0.09 standard deviation increase in social–emotional health outcomes. This effect size is considerably smaller than the 0.41 standard deviation typically considered practically significant [75]. However, this association is only for an all‐for‐one substitution. One‐for‐one substitution, which can be examined using compositional isotemporal substitution modeling [76], may demonstrate a stronger association; for example, increasing engagement in MVPA solely at the expense of time spent sedentary. The association between 24‐h movement behaviors and social–emotional health was relatively consistent across outcomes, including internalizing symptoms, externalizing symptoms, social functioning, and positive mental health, suggesting that the benefits of engaging in healthier 24‐h movement behavior composition may be relatively universal across indicators of social–emotional health. The results of the subgroup analysis demonstrated that the association between sleep and social–emotional health was stronger among adolescents compared to children. Adolescence is a period of rapid brain development characterized by declines in volumes of cortical gray matter [77] which may be associated with sleep patterns [78]. These differences in gray matter volume may help explain why sleep is more strongly associated with social–emotional health in adolescents [79], which might be a reason that the association was stronger in adolescents compared to younger children. Another possible explanation is that adolescents may begin to have greater autonomy over their sleep, which may impact their sleep patterns [80]. Adolescents, especially mature adolescents aged 15 years and older, may experience changes to their circadian rhythms and homeostatic sleep/wake timing towards later times, which may not align with social and scholastic obligations, contributing to increased sleep deprivation [81].

It is important to note that most of the studies which assessed social–emotional outcomes were cross‐sectional. Therefore, it is possible that the results could reflect reverse causality. For example, emotional problems may cause sleep disturbances among children [82], while children experiencing internalizing symptoms might disengage from physical activity and engage in more sedentary time [83]. Interestingly, while movement behaviors were related to social emotional health cross‐sectionally, these relationships were often not observed longitudinally. For example, Chong, Parrish, Cliff, Dumuid, Okely [57] showed that spending more time sleeping and less time sedentary and in LPA was cross‐sectionally related to fewer internalizing problems in adolescents, associations which were not observed longitudinally. Similarly, Taylor, Haszard, Meredith‐Jones, Azeem, Galland, Heath, Taylor, Healey [68] found that spending more time in MVPA relative to other movement behaviors was cross‐sectionally but not longitudinally related to fewer anxiety symptoms and more resilience in five‐year‐old children. Further, Tan et al. [67] showed that LPA was cross‐sectionally associated with self‐esteem and MVPA was cross‐sectionally related to relationship with friends in 10‐year‐old children; however, neither had a significant longitudinal relationship. Only a single study reported that spending more time in MVPA relative to other movement behaviors was cross‐sectionally and longitudinally related to fewer internalizing symptoms in 10‐year‐old children [55]. Therefore, more longitudinal studies are needed to determine the directionality of association and strengthen the evidence base for the association between 24‐h movement behavior composition and social–emotional health in children and adolescents.

Interestingly, no component of 24‐h movement behaviors was associated with cognitive development in children and adolescents. This finding is in conflict with the results from another review which reported that achieving individual components of the 24‐h movement behavior guidelines and the overall guidelines was positively associated with academic achievement and cognitive functioning [84]. However, that review only included studies of children and adolescents aged 5–18 years. In the current review, 8 of the 11 studies that assessed cognitive outcomes were in children younger than 5 years of age, making direct comparisons difficult. There is clearly a need for more studies among children and adolescents to determine the association between 24‐h movement behavior compositions and cognitive development. Although not significant, results from the meta‐analysis did point towards a trend that spending more time sedentary and less time in LPA may be associated with better cognitive outcomes, especially when considering inhibition as the outcome. While these results may seem initially surprising, there are plausible mechanisms to explain these findings. For example, previous studies have demonstrated that engaging in cognitively engaging activities, typically performed while sedentary, such as reading or playing video games, is favorably associated with cognitive development in children and youth [4, 85, 86]. Additionally, it may be important to consider that the majority of children's LPA and sedentary time occurs while at school [87, 88, 89]. Previous research has demonstrated that children who spend more time being sedentary during school time have fewer hyperactivity and inattention symptoms [90] and children who spend more time in sedentary activities relative to other movement behaviors in childcare exhibit better psychosocial functioning [91]. Similarly, research in preschool children demonstrated that average acceleration recorded using accelerometers has a strong negative association with children's inhibitory control and attention as reported by early childhood educators [92]. It is possible that poor inhibitory control could manifest as increased engagement in LPA at the expense of sedentary behaviors in these settings, for example, if a child finds it difficult to remain seated for an entire class. Variation in associations across contexts may partly explain the null findings, as the association between some components of 24‐h movement behavior compositions may conflict depending on specific contexts such as between school and home environments. Future studies are warranted to determine if there are time‐varying effects, exploring how 24‐h movement behaviors at different times of the day and in different contexts (e.g., during class, after school) are related to cognitive development during childhood and adolescence.

To our knowledge, this is the first study to conduct a meta‐analysis on 24‐h movement behavior compositional data and emotional and cognitive health in children and adolescents. The results provide the most up‐to‐date evidence into the relative association between each component of 24‐h movement behaviors and social–emotional health and cognitive development in children and adolescents. The study has several strengths, including conducting a comprehensive systematic review supplemented by manual searches to identify all published studies relevant to the research question. Additionally, this study used machine learning software to assist with screening titles and abstracts, which can decrease the error rate in screening due to being presented in a random order [93]. Further, unlike previous reviews that have taken descriptive approaches to synthesizing compositional data, a meta‐analysis was undertaken, resulting in the reporting of direction and strength of associations. There are a few areas that require future research attention. No studies have compared the association between boys and girls. Given potential biological and societal factors impacting experiences, research comparing boys and girls could provide important insights into the association between 24‐h movement behaviors and mental health. Additionally, two studies reported on screen time separate from device‐assessed sedentary time. More research into screen time specifically could provide important insights into how different types of sedentary activities as part of a 24‐h time use composition relate to mental health.

This study also had several limitations that need to be considered. First, most studies included in the meta‐analyses reported on cross‐sectional data; therefore, the results from the present study cannot be used to infer causation, and it is not possible to rule out reverse causation. Second, only studies written in English were eligible for inclusion in this study; therefore, relevant articles may have been excluded if they were written in languages other than English. Additionally, there was some between‐study heterogeneity observed in the meta‐analyses; however, given the relatively limited number of studies included in each meta‐analysis, and the need for a relatively large number of studies to conduct subgroup analysis using a robust variance estimator, it was not feasible to conduct a meta‐regression to examine all sources of heterogeneity. More studies in diverse populations, including diverse social–emotional and cognitive outcomes, are necessary to examine differences based on possible effect modifiers. Lastly, only five of the included studies (reporting on three unique samples) recruited samples representative of national populations, potentially limiting the generalizability of the findings. Most studies were conducted in urban areas and recruited children from generalist schools and childcare centers. Therefore, certain groups of children, such as those living in rural areas or children with disabilities, may not have been adequately represented, or even purposefully excluded from studies included in the meta‐analyses.

## 5 Conclusion

This paper synthesized the results from studies using compositional data analysis to examine the association between 24‐h movement behaviors and social–emotional health and cognitive development outcomes in children and adolescents. The results showed that replacing sedentary time with MVPA or sleep may promote social–emotional health in children and adolescents. Interestingly, based on available evidence, no components of the 24‐h movement behavior composition were significantly related to cognitive development. However, most existing evidence is from cross‐sectional studies; evidence from longitudinal studies is less conclusive. More longitudinal studies are needed to strengthen the evidence base. Additionally, further research is warranted to better understand the optimal 24‐h movement behavior combinations, including different types of physical activity and sedentary behaviors (e.g., cognitively active, screen‐based), for children and adolescents' social–emotional health and cognitive development.

## Author Contributions

Conceptualization: M.B. Methodology: M.B. Formal analysis: M.B. Investigation: M.B., H.F.W.W. and K.F. Data curation: M.B., H.F.W.W. and K.F. Writing – original draft: M.B. Writing – review and editing: H.F.W.W., K.F., G.T., M.O., S.K.M., S.R.G., T.A., S.G.T., B.A.B., S.M.P., L.V., P.T., K.D.H., M.Y.W.K. and J.C.

## Ethics Statement

The current study used publicly available data and therefore was exempt from ethics approval or written informed consent procedures.

## Conflicts of Interest

The authors declare no conflicts of interest.

## Supporting information


**Appendix A1:** sms70120‐sup‐0001‐AppendixA1.docx.

**Appendix B1:** supinfo/sms70120‐sup‐0002‐AppendixB1.docx.

**Appendix C1:** supinfo/sms70120‐sup‐0003‐AppendixC1.docx.

**Appendix D1:** supinfo/sms70120‐sup‐0004‐AppendixD1.docx.

**Data S1:** sms70120‐sup‐0005‐DataS1.xlsx.

## Data Availability

The data necessary to complete this analysis are publicly available in the original publications included in the review, or from the authors of the original publications upon request. The code to process the data are available from the lead author upon request.

## References

[1] M. S. Tremblay , V. Carson , J.‐P. Chaput , et al., “Canadian 24‐Hour Movement Guidelines for Children and Youth: An Integration of Physical Activity, Sedentary Behaviour, and Sleep,” Applied Physiology, Nutrition, and Metabolism 41, no. 6 Suppl 3 (2016): S311–S327. 10.1139/apnm-2016-0151 27306437

[2] M. S. Tremblay , J.‐P. Chaput , K. B. Adamo , et al., “Canadian 24‐Hour Movement Guidelines for the Early Years (0–4 Years): An Integration of Physical Activity, Sedentary Behaviour, and Sleep,” BMC Public Health 17, no. 5 (2017): 874. 29219102 10.1186/s12889-017-4859-6 PMC5773896

[3] M. S. Tremblay , “Introducing 24‐Hour Movement Guidelines for the Early Years: A New Paradigm Gaining Momentum,” Journal of Physical Activity & Health 17, no. 1 (2020): 92–95. 31711035 10.1123/jpah.2019-0401

[4] V. Carson , S. Hunter , N. Kuzik , et al., “Systematic Review of Sedentary Behaviour and Health Indicators in School‐Aged Children and Youth: An Update,” Applied Physiology, Nutrition, and Metabolism 41, no. 6 Suppl 3 (2016): S240–S265. 10.1139/apnm-2015-0630 27306432

[5] V. J. Poitras , C. E. Gray , M. M. Borghese , et al., “Systematic Review of the Relationships Between Objectively Measured Physical Activity and Health Indicators in School‐Aged Children and Youth,” Applied Physiology, Nutrition, and Metabolism 41, no. 6 Suppl 3 (2016): S197–S239. 10.1139/apnm-2015-0663 27306431

[6] J.‐P. Chaput , C. E. Gray , V. J. Poitras , et al., “Systematic Review of the Relationships Between Sleep Duration and Health Indicators in School‐Aged Children and Youth,” Applied Physiology, Nutrition, and Metabolism 41, no. 6 Suppl 3 (2016): S266–S282. 10.1139/apnm-2015-0627 27306433

[7] V. Carson , E.‐Y. Lee , L. Hewitt , et al., “Systematic Review of the Relationships Between Physical Activity and Health Indicators in the Early Years (0‐4 Years),” BMC Public Health 17, no. 5 (2017): 854. 29219090 10.1186/s12889-017-4860-0 PMC5753397

[8] V. J. Poitras , C. E. Gray , X. Janssen , et al., “Systematic Review of the Relationships Between Sedentary Behaviour and Health Indicators in the Early Years (0–4 Years),” BMC Public Health 17, no. 5 (2017): 868. 29219092 10.1186/s12889-017-4849-8 PMC5773886

[9] J.‐P. Chaput , C. E. Gray , V. J. Poitras , et al., “Systematic Review of the Relationships Between Sleep Duration and Health Indicators in the Early Years (0–4 Years),” BMC Public Health 17, no. 5 (2017): 855. 29219078 10.1186/s12889-017-4850-2 PMC5773910

[10] T. J. Saunders , C. E. Gray , V. J. Poitras , et al., “Combinations of Physical Activity, Sedentary Behaviour and Sleep: Relationships With Health Indicators in School‐Aged Children and Youth,” Applied Physiology, Nutrition, and Metabolism 41, no. 6 Suppl 3 (2016): S283–S293. 10.1139/apnm-2015-0626 27306434

[11] N. Kuzik , V. J. Poitras , M. S. Tremblay , E.‐Y. Lee , S. Hunter , and V. Carson , “Systematic Review of the Relationships Between Combinations of Movement Behaviours and Health Indicators in the Early Years (0‐4 Years),” BMC Public Health 17, no. 5 (2017): 849. 29219071 10.1186/s12889-017-4851-1 PMC5773877

[12] Z. Pedišić , D. Dumuid , and T. A. Olds , “Integrating Sleep, Sedentary Behaviour, and Physical Activity Research in the Emerging Field of Time‐Use Epidemiology: Definitions, Concepts, Statistical Methods, Theoretical Framework, and Future Directions,” Kinesiology 49 (2017): 252–269.

[13] D. Dumuid , Ž. Pedišić , J. Palarea‐Albaladejo , J. A. Martín‐Fernández , K. Hron , and T. Olds , “Compositional Data Analysis in Time‐Use Epidemiology: What, Why, How,” International Journal of Behavioral Nutrition and Physical Activity 17, no. 7 (2020): 2220, 10.3390/ijerph17072220. PMC7177981 32224966

[14] S. F. M. Chastin , J. Palarea‐Albaladejo , M. L. Dontje , and D. A. Skelton , “Combined Effects of Time Spent in Physical Activity, Sedentary Behaviors and Sleep on Obesity and Cardio‐Metabolic Health Markers: A Novel Compositional Data Analysis Approach,” PLoS One 10, no. 10 (2015): e0139984. 26461112 10.1371/journal.pone.0139984 PMC4604082

[15] D. Dumuid , T. E. Stanford , J.‐A. Martin‐Fernández , et al., “Compositional Data Analysis for Physical Activity, Sedentary Time and Sleep Research,” Statistical Methods in Medical Research 27, no. 12 (2017): 3726–3738. 28555522 10.1177/0962280217710835

[16] R. A. Mekary , W. C. Willett , F. B. Hu , and E. L. Ding , “Isotemporal Substitution Paradigm for Physical Activity Epidemiology and Weight Change,” American Journal of Epidemiology 170, no. 4 (2009): 519–527. 19584129 10.1093/aje/kwp163 PMC2733862

[17] H. A. Whiteford , L. Degenhardt , J. Rehm , et al., “Global Burden of Disease Attributable to Mental and Substance Use Disorders: Findings From the Global Burden of Disease Study 2010,” Lancet 382, no. 9904 (2013): 1575–1586. 23993280 10.1016/S0140-6736(13)61611-6

[18] M. K. Nock , A. E. Kazdin , E. Hiripi , and R. C. Kessler , “Lifetime Prevalence, Correlates, and Persistence of Oppositional Defiant Disorder: Results From the National Comorbidity Survey Replication,” Journal of Child Psychology and Psychiatry 48, no. 7 (2007): 703–713. 17593151 10.1111/j.1469-7610.2007.01733.x

[19] World Health Organization , “Mental Health,” 2024, https://www.who.int/health‐topics/mental‐health#tab=tab_1.

[20] World Health Organization , “Comprehensive Mental Heath Action Plan 2013–2030.” 2021, https://www.who.int/publications/i/item/9789240031029.

[21] G. F. Bauer , O. Hämmig , and C. L. Keyes , “Mental Health as a Complete State: How the Salutogenic Perspective Completes the Picture,” in Bridging Occupational, Organizational and Public Health: A Transdisciplinary Approach, ed. G. F. Bauer and O. Hämmig (Springer Netherlands, 2014), 179–192.

[22] M. Rodriguez‐Ayllon , C. Cadenas‐Sánchez , F. Estévez‐López , et al., “Role of Physical Activity and Sedentary Behavior in the Mental Health of Preschoolers, Children and Adolescents: A Systematic Review and Meta‐Analysis,” Sports Medicine 49, no. 9 (2019): 1383–1410. 30993594 10.1007/s40279-019-01099-5

[23] C. Álvarez‐Bueno , C. Pesce , I. Cavero‐Redondo , M. Sánchez‐López , J. A. Martínez‐Hortelano , and V. Martínez‐Vizcaíno , “The Effect of Physical Activity Interventions on Children's Cognition and Metacognition: A Systematic Review and Meta‐Analysis,” Journal of the American Academy of Child and Adolescent Psychiatry 56, no. 9 (2017): 729–738. 28838577 10.1016/j.jaac.2017.06.012

[24] J. Zhang , S. X. Yang , L. Wang , L. H. Han , and X. Y. Wu , “The Influence of Sedentary Behaviour on Mental Health Among Children and Adolescents: A Systematic Review and Meta‐Analysis of Longitudinal Studies,” Journal of Affective Disorders 306 (2022): 90–114. 35304232 10.1016/j.jad.2022.03.018

[25] S. Li , J. Guo , K. Zheng , M. Shi , and T. Huang , “Is Sedentary Behavior Associated With Executive Function in Children and Adolescents? A Systematic Review,” Frontiers in Public Health 10 (2022): 832845. 35186852 10.3389/fpubh.2022.832845 PMC8847290

[26] C. Marino , B. Andrade , S. C. Campisi , et al., “Association Between Disturbed Sleep and Depression in Children and Youths: A Systematic Review and Meta‐Analysis of Cohort Studies,” JAMA Network Open 4, no. 3 (2021): e212373. 33749768 10.1001/jamanetworkopen.2021.2373 PMC7985724

[27] R. G. Astill , K. B. van der Heijden , M. H. van Ijzendoorn , and E. J. W. van Someren , “Sleep, Cognition, and Behavioral Problems in School‐Age Children: A Century of Research Meta‐Analyzed,” Psychological Bulletin 138, no. 6 (2012): 1109–1138. 22545685 10.1037/a0028204

[28] M. A. Short , S. A. Booth , O. Omar , L. Ostlundh , and T. Arora , “The Relationship Between Sleep Duration and Mood in Adolescents: A Systematic Review and Meta‐Analysis,” Sleep Medicine Reviews 52 (2020): 101311. 32240932 10.1016/j.smrv.2020.101311

[29] S. Zahran , C. Visser , A. Ross‐White , and I. Janssen , “A Systematic Review of Compositional Analysis Studies Examining the Associations Between Sleep, Sedentary Behaviour, and Physical Activity With Health Indicators in Early Childhood,” Journal of Activity, Sedentary and Sleep Behaviors 2 (2023): 1. 40217382 10.1186/s44167-022-00012-2 PMC11960365

[30] S. Rollo , O. Antsygina , and M. S. Tremblay , “The Whole Day Matters: Understanding 24‐Hour Movement Guideline Adherence and Relationships With Health Indicators Across the Lifespan,” Journal of Sport and Health Science 9, no. 6 (2020): 493–510. 32711156 10.1016/j.jshs.2020.07.004 PMC7749249

[31] H. Sampasa‐Kanyinga , I. Colman , G. S. Goldfield , et al., “Combinations of Physical Activity, Sedentary Time, and Sleep Duration and Their Associations With Depressive Symptoms and Other Mental Health Problems in Children and Adolescents: A Systematic Review,” International Journal of Behavioral Nutrition and Physical Activity 17, no. 1 (2020): 72. 32503638 10.1186/s12966-020-00976-x PMC7273653

[32] R. Dale , T. O'Rourke , B. Nussbaumer‐Streit , and T. Probst , “24‐Hour Movement Behaviours and Mental Health in Non‐Clinical Populations: A Systematic Review,” PLoS One 20, no. 6 (2025): e0325445. 40489524 10.1371/journal.pone.0325445 PMC12148175

[33] N. Kuzik , M. J. Duncan , N. Beshara , M. MacDonald , D. A. S. Silva , and M. S. Tremblay , “A Systematic Review and Meta‐Analysis of the First Decade of Compositional Data Analyses of 24‐Hour Movement Behaviours, Health, and Well‐Being in School‐Aged Children,” Journal of Activity, Sedentary and Sleep Behaviors 4, no. 1 (2025): 4. 40217545 10.1186/s44167-025-00076-w PMC11948812

[34] R. S. Falck , J. C. Davis , L. Li , E. Stamatakis , and T. Liu‐Ambrose , “Preventing the ‘24‐Hour Babel’: The Need for a Consensus on a Consistent Terminology Scheme for Physical Activity, Sedentary Behaviour and Sleep,” British Journal of Sports Medicine 56, no. 7 (2022): 367–368. 34556466 10.1136/bjsports-2021-104487 PMC8938678

[35] M. J. Page , J. E. McKenzie , P. M. Bossuyt , et al., “The PRISMA 2020 Statement: An Updated Guideline for Reporting Systematic Reviews,” BMJ (Clinical Research Ed.) 372 (2021): n71. 10.1136/bmj.n71 PMC8005924 33782057

[36] World Health Organization , “Guidelines on Physical Activity, Sedentary Behaviour and Sleep for Children Under 5 Years of Age,” 2019, https://www.who.int/publications/i/item/9789241550536. 31091057

[37] S. M. Sawyer , P. S. Azzopardi , D. Wickremarathne , and G. C. Patton , “The Age of Adolescence,” Lancet Child & Adolescent Health 2, no. 3 (2018): 223–228. 30169257 10.1016/S2352-4642(18)30022-1

[38] R. Ross , J.‐P. Chaput , L. M. Giangregorio , et al., “Canadian 24‐Hour Movement Guidelines for Adults Aged 18–64 Years and Adults Aged 65 Years or Older: An Integration of Physical Activity, Sedentary Behaviour, and Sleep,” Applied Physiology, Nutrition, and Metabolism 45, no. 10 Suppl 2 (2020): S57–S102. 10.1139/apnm-2020-0467 33054332

[39] R. van de Schoot , J. de Bruin , R. Schram , et al., “An Open Source Machine Learning Framework for Efficient and Transparent Systematic Reviews,” Nature Machine Intelligence 3, no. 2 (2021): 125–133.

[40] M. Bourke , A. Haddara , A. Loh , V. Carson , B. Breau , and P. Tucker , “Adherence to the World Health Organization's Physical Activity Recommendation in Preschool‐Aged Children: A Systematic Review and Meta‐Analysis of Accelerometer Studies,” International Journal of Behavioral Nutrition and Physical Activity 20, no. 1 (2023): 52. 37101226 10.1186/s12966-023-01450-0 PMC10132436

[41] M. J. Downes , M. L. Brennan , H. C. Williams , and R. S. Dean , “Development of a Critical Appraisal Tool to Assess the Quality of Cross‐Sectional Studies (AXIS),” BMJ Open 6, no. 12 (2016): e011458. 10.1136/bmjopen-2016-011458 PMC5168618 27932337

[42] D. Rorem , V. E. Ezeugwu , V. J. Joly , et al., “Finding the Balance: The Influence of Movement Behaviours on Childhood Behaviour Problems,” Mental Health and Physical Activity 26 (2024): 100593.

[43] F. Li , L. Yin , W. Luo , et al., “Isotemporal Substitution Effect of 24‐Hour Movement Behavior on the Mental Health of Chinese Preschool Children,” Frontiers in Public Health 12 (2024): 1288262. 38560447 10.3389/fpubh.2024.1288262 PMC10979542

[44] V. Carson , M. S. Tremblay , J.‐P. Chaput , and S. F. M. Chastin , “Associations Between Sleep Duration, Sedentary Time, Physical Activity, and Health Indicators Among Canadian Children and Youth Using Compositional Analyses,” Applied Physiology, Nutrition, and Metabolism 41, no. 6 Suppl 3 (2016): S294–S302. 10.1139/apnm-2016-0026 27306435

[45] M. Rodríguez‐Barranco , A. Tobías , D. Redondo , E. Molina‐Portillo , and M. J. Sánchez , “Standardizing Effect Size From Linear Regression Models With Log‐Transformed Variables for Meta‐Analysis,” BMC Medical Research Methodology 17, no. 1 (2017): 44. 28302052 10.1186/s12874-017-0322-8 PMC5356327

[46] L. V. Hedges , E. Tipton , and M. C. Johnson , “Robust Variance Estimation in Meta‐Regression With Dependent Effect Size Estimates,” Research Synthesis Methods 1, no. 1 (2010): 39–65. 26056092 10.1002/jrsm.5

[47] W. Viechtbauer , “Conducting Meta‐Analyses in R With the Metafor Package,” Journal of Statistical Software 36, no. 3 (2010): 1–48.

[48] J. Pustejovsky , “clubSandwich: Cluster‐Robust (sandwich) Variance Estimators With Small‐Sample Corrections,” 2014, https://cran.r‐project.org/web/packages/clubSandwich/index.html.

[49] J. E. Pustejovsky and E. Tipton , “Meta‐Analysis With Robust Variance Estimation: Expanding the Range of Working Models,” Prevention Science 23, no. 3 (2022): 425–438. 33961175 10.1007/s11121-021-01246-3

[50] E. Tipton , “Small Sample Adjustments for Robust Variance Estimation With Meta‐Regression,” Psychological Methods 20, no. 3 (2015): 375–393. 24773356 10.1037/met0000011

[51] M. Harrer , P. Cuijpers , T. Furukawa , and D. E. Ebert , “dmetar: Companion R Package for The Guide ‘Doing Meta‐Analysis in R’,” 2019, https://dmetar.protectlab.org/.

[52] H. Wickham , ggplot2: Elegant Graphics for Data Analysis (Springer‐Verlag, 2016).

[53] A. Kassambara , “ggpubr: ‘ggplot2’ Based Publication Ready Plots,” 2023, https://CRAN.R‐project.org/package=ggpubr.

[54] M. A. Rodgers and J. E. Pustejovsky , “Evaluating Meta‐Analytic Methods to Detect Selective Reporting in the Presence of Dependent Effect Sizes,” Psychological Methods 26, no. 2 (2021): 141–160. 32673040 10.1037/met0000300

[55] M. Bourke , T. Alsop , R. L. Peters , et al., “The Cross‐Sectional and Longitudinal Association Between 24‐Hour Movement Behavior Compositions With Body Mass Index, Waist Circumference, and Internalizing and Externalizing Symptoms in 6‐Year‐Old Children,” Journal of Physical Activity and Health 22 (2024): 1–13. 39547218 10.1123/jpah.2024-0482

[56] T. A. Bezerra , C. C. T. Clark , A. N. Souza Filho , et al., “24‐Hour Movement Behaviour and Executive Function in Preschoolers: A Compositional and Isotemporal Reallocation Analysis,” European Journal of Sport Science 21, no. 7 (2021): 1064–1072. 32654601 10.1080/17461391.2020.1795274

[57] K. H. Chong , A. M. Parrish , D. P. Cliff , D. Dumuid , and A. D. Okely , “Cross‐Sectional and Longitudinal Associations Between 24‐Hour Movement Behaviours, Recreational Screen Use and Psychosocial Health Outcomes in Children: A Compositional Data Analysis Approach,” International Journal of Environmental Research and Public Health 18, no. 11 (2021): 5995. 34204928 10.3390/ijerph18115995 PMC8199728

[58] F. R. de Faria , D. Barbosa , C. A. Howe , K. L. R. Canabrava , J. E. Sasaki , and P. R. Dos Santos Amorim , “Time‐Use Movement Behaviors Are Associated With Scores of Depression/Anxiety Among Adolescents: A Compositional Data Analysis,” PLoS One 17, no. 12 (2022): e0279401. 36584176 10.1371/journal.pone.0279401 PMC9803290

[59] S. J. Fairclough , R. Tyler , J. R. Dainty , et al., “Cross‐Sectional Associations Between 24‐Hour Activity Behaviours and Mental Health Indicators in Children and Adolescents: A Compositional Data Analysis,” Journal of Sports Sciences 39, no. 14 (2021): 1602–1614. 33615990 10.1080/02640414.2021.1890351

[60] N. Kuzik , P. J. Naylor , J. C. Spence , and V. Carson , “Movement Behaviours and Physical, Cognitive, and Social‐Emotional Development in Preschool‐Aged Children: Cross‐Sectional Associations Using Compositional Analyses,” PLoS One 15, no. 8 (2020): e0237945. 32810172 10.1371/journal.pone.0237945 PMC7433874

[61] P. W. C. Lau , H. Song , D. Song , et al., “24‐Hour Movement Behaviors and Executive Functions in Preschoolers: A Compositional and Isotemporal Reallocation Analysis,” Child Development 95, no. 2 (2024): e110–e121. 37787120 10.1111/cdev.14013

[62] Z. Lu , X. Qu , J. Chang , et al., “Reallocation of Time Between Preschoolers' 24‐h Movement Behaviours and Executive Functions: A Compositional Data Analysis,” Journal of Sports Sciences 41, no. 12 (2023): 1187–1195. 37724814 10.1080/02640414.2023.2260632

[63] B. Lu , Z. Huang , J. Lou , R. Li , and Y. Zhou , “Associations Between 24‐Hour Activity Behaviours and Emotional and Behavioural Problems of Left‐Behind Children: A Component Analysis of Data From a Cross‐Sectional Study,” BMJ Open 14, no. 8 (2024): e084749. 10.1136/bmjopen-2024-084749 PMC11367308 39645272

[64] E. Ng , M. Wake , T. Olds , et al., “Equivalence Curves for Healthy Lifestyle Choices,” Pediatrics 147, no. 4 (2021): e2020025395. 33771915 10.1542/peds.2020-025395

[65] N. Padmapriya , J. Y. Bernard , S. Y. X. Tan , et al., “The Prospective Associations of 24‐Hour Movement Behaviors and Domain‐Specific Activities With Executive Function and Academic Achievement Among School‐Aged Children in Singapore,” Frontiers in Public Health 12 (2024): 1412634. 39296832 10.3389/fpubh.2024.1412634 PMC11409845

[66] C. W. St. Laurent , C. L. Rasmussen , J. F. Holmes , et al., “Associations of Activity, Sedentary, and Sleep Behaviors With Cognitive and Social‐Emotional Health in Early Childhood,” Journal of Activity, Sedentary and Sleep Behaviors 2, no. 1 (2023): 7. 38798902 10.1186/s44167-023-00016-6 PMC11116218

[67] S. Y. X. Tan , N. Padmapriya , J. Y. Bernard , et al., “Cross‐Sectional and Prospective Associations Between Children's 24‐h Time Use and Their Health‐Related Quality of Life: A Compositional Isotemporal Substitution Approach,” Lancet Regional Health. Western Pacific 41 (2023): 100918. 37842643 10.1016/j.lanwpc.2023.100918 PMC10570705

[68] R. W. Taylor , J. J. Haszard , K. A. Meredith‐Jones , et al., “Associations Between Activity, Sedentary and Sleep Behaviours and Psychosocial Health in Young Children: A Longitudinal Compositional Time‐Use Study,” Journal of Activity, Sedentary and Sleep Behaviors 2, no. 1 (2023): 3. 40217436 10.1186/s44167-022-00011-3 PMC11960262

[69] A. Watson , D. Dumuid , and T. Olds , “Associations Between 24‐Hour Time Use and Academic Achievement in Australian Primary School–Aged Children,” Health Education & Behavior 47, no. 6 (2020): 905–913. 32844698 10.1177/1090198120952041

[70] S. Zahran , D. P. Cliff , D. Antczak , et al., “Optimal Levels of Sleep, Sedentary Behaviour, and Physical Activity Needed to Support Cognitive Function in Children of the Early Years,” BMC Pediatrics 24, no. 1 (2024): 735. 39543525 10.1186/s12887-024-05186-z PMC11562365

[71] J. J. Haszard , K. Meredith‐Jones , V. Farmer , S. Williams , B. Galland , and R. Taylor , “Non‐Wear Time and Presentation of Compositional 24‐Hour Time‐Use Analyses Influence Conclusions About Sleep and Body Mass Index in Children,” Journal for the Measurement of Physical Behaviour 3, no. 3 (2020): 204–210.

[72] S. J. H. Biddle , S. Ciaccioni , G. Thomas , and I. Vergeer , “Physical Activity and Mental Health in Children and Adolescents: An Updated Review of Reviews and an Analysis of Causality,” Psychology of Sport and Exercise 42 (2019): 146–155.

[73] A. Kandola , G. Lewis , D. P. J. Osborn , B. Stubbs , and J. F. Hayes , “Device‐Measured Sedentary Behaviour and Anxiety Symptoms During Adolescence: A 6‐Year Prospective Cohort Study,” Psychological Medicine 52, no. 14 (2022): 2962–2971. 33336634 10.1017/S0033291720004948 PMC9693656

[74] V. Bacaro , K. Miletic , and E. Crocetti , “A Meta‐Analysis of Longitudinal Studies on the Interplay Between Sleep, Mental Health, and Positive Well‐Being in Adolescents,” International Journal of Clinical and Health Psychology 24, no. 1 (2024): 100424. 38125984 10.1016/j.ijchp.2023.100424 PMC10730350

[75] C. J. Ferguson , “An Effect Size Primer: A Guide for Clinicians and Researchers,” Professional Psychology: Research and Practice 40 (2016): 532–538.

[76] D. Dumuid , Ž. Pedišić , T. E. Stanford , et al., “The Compositional Isotemporal Substitution Model: A Method for Estimating Changes in a Health Outcome for Reallocation of Time Between Sleep, Physical Activity and Sedentary Behaviour,” Statistical Methods in Medical Research 28, no. 3 (2017): 846–857. 29157152 10.1177/0962280217737805

[77] T. Paus , M. Keshavan , and J. N. Giedd , “Why Do Many Psychiatric Disorders Emerge During Adolescence?,” Nature Reviews. Neuroscience 9, no. 12 (2008): 947–957. 19002191 10.1038/nrn2513 PMC2762785

[78] C. Dutil , J. J. Walsh , R. B. Featherstone , et al., “Influence of Sleep on Developing Brain Functions and Structures in Children and Adolescents: A Systematic Review,” Sleep Medicine Reviews 42 (2018): 184–201. 30241996 10.1016/j.smrv.2018.08.003

[79] W. Cheng , E. Rolls , W. Gong , et al., “Sleep Duration, Brain Structure, and Psychiatric and Cognitive Problems in Children,” Molecular Psychiatry 26, no. 8 (2021): 3992–4003. 32015467 10.1038/s41380-020-0663-2 PMC8855973

[80] S. M. Tashjian , J. L. Mullins , and A. Galván , “Bedtime Autonomy and Cellphone Use Influence Sleep Duration in Adolescents,” Journal of Adolescent Health 64, no. 1 (2019): 124–130. 10.1016/j.jadohealth.2018.07.018 30366713

[81] S. J. Crowley , C. Acebo , and M. A. Carskadon , “Sleep, Circadian Rhythms, and Delayed Phase in Adolescence,” Sleep Medicine 8, no. 6 (2007): 602–612. 17383934 10.1016/j.sleep.2006.12.002

[82] N. Lovato and M. Gradisar , “A Meta‐Analysis and Model of the Relationship Between Sleep and Depression in Adolescents: Recommendations for Future Research and Clinical Practice,” Sleep Medicine Reviews 18, no. 6 (2014): 521–529. 24857255 10.1016/j.smrv.2014.03.006

[83] K. E. Gunnell , M. F. Flament , A. Buchholz , et al., “Examining the Bidirectional Relationship Between Physical Activity, Screen Time, and Symptoms of Anxiety and Depression Over Time During Adolescence,” Preventive Medicine 88 (2016): 147–152. 27090920 10.1016/j.ypmed.2016.04.002

[84] R. Bao , H. Qin , A. R. Memon , et al., “Is Adherence to the 24‐h Movement Guidelines Associated With Greater Academic‐Related Outcomes in Children and Adolescents? A Systematic Review and Meta‐Analysis,” European Journal of Pediatrics 183, no. 5 (2024): 2003–2014. 38416259 10.1007/s00431-024-05461-2

[85] C. Cristi‐Montero , S. Hernandez‐Jaña , J. P. Zavala‐Crichton , et al., “Mentally Active but Not Inactive Sedentary Behaviors Are Positively Related to Adolescents' Cognitive‐Academic Achievements, a Cross‐Sectional Study—The Cogni‐Action Project,” Mental Health and Physical Activity 25 (2023): 100561.

[86] T. Sanders , M. Noetel , P. Parker , et al., “An Umbrella Review of the Benefits and Risks Associated With Youths' Interactions With Electronic Screens,” Nature Human Behaviour 8, no. 1 (2024): 82–99. 10.1038/s41562-023-01712-8 37957284

[87] D. P. Bailey , S. J. Fairclough , L. A. Savory , et al., “Accelerometry‐Assessed Sedentary Behaviour and Physical Activity Levels During the Segmented School Day in 10–14‐Year‐Old Children: The HAPPY Study,” European Journal of Pediatrics 171, no. 12 (2012): 1805–1813. 22983026 10.1007/s00431-012-1827-0

[88] G. McLellan , R. Arthur , S. Donnelly , and D. S. Buchan , “Segmented Sedentary Time and Physical Activity Patterns Throughout the Week From Wrist‐Worn ActiGraph GT3X+ Accelerometers Among Children 7–12 Years Old,” Journal of Sport and Health Science 9, no. 2 (2020): 179–188. 32099726 10.1016/j.jshs.2019.02.005 PMC7031810

[89] R. M. Tassitano , R. G. Weaver , M. C. M. Tenório , K. Brazendale , and M. W. Beets , “Physical Activity and Sedentary Time of Youth in Structured Settings: A Systematic Review and Meta‐Analysis,” International Journal of Behavioral Nutrition and Physical Activity 17, no. 1 (2020): 160. 33276782 10.1186/s12966-020-01054-y PMC7716454

[90] B. G. G. da Costa , B. Bruner , G. H. Raymer , et al., “Association of Daily and Time‐Segmented Physical Activity and Sedentary Behaviour With Mental Health of School Children and Adolescents From Rural Northeastern Ontario, Canada,” Frontiers in Psychology 13 (2022): 1025444. 36389567 10.3389/fpsyg.2022.1025444 PMC9644206

[91] M. Bourke , L. M. Vanderloo , J. D. Irwin , et al., “Association Between Childcare Movement Behaviour Compositions With Health and Development Among Preschoolers: Finding the Optimal Combinations of Physical Activities and Sedentary Time,” Journal of Sports Sciences 40, no. 18 (2022): 2085–2094. 36227866 10.1080/02640414.2022.2134969

[92] A. E. Koepp and E. T. Gershoff , “Leveraging an Intensive Time Series of Young Children's Movement to Capture Impulsive and Inattentive Behaviors in a Preschool Setting,” Child Development 95 (2024): 1641–1658. 38655639 10.1111/cdev.14100 PMC11499294

[93] Z. Wang , T. Nayfeh , J. Tetzlaff , P. O'Blenis , and M. H. Murad , “Error Rates of Human Reviewers During Abstract Screening in Systematic Reviews,” PLoS One 15, no. 1 (2020): e0227742. 31935267 10.1371/journal.pone.0227742 PMC6959565

