# Supplementary material for: Association Between 24‐h Movement Behaviors and Mental Health in Children and Adolescents: A Systematic Review and Compositional Data Meta‐Analysis
Source: Scand J Med Sci Sports. 2025 Aug 19;35(8):e70120. doi: 10.1111/sms.70120 (PMC12363385; doi:10.1111/sms.70120)
Supplement: Supplementary file 1 — Appendix A1: sms70120‐sup‐0001‐AppendixA1.docx. [file SMS-35-e70120-s001.docx]

**Appendix A**

*Search terms*

**Table A1**

*Systematic search terms*

|  |  |
| --- | --- |
| 1 | 24-hour OR movement behavio?r* OR physical behavio?r* OR time-use OR composition* data OR composition* analys* OR (physical activity AND sleep AND sedentary) |
| 2 | mental health OR depress* OR anxiety OR stress OR self-esteem OR self-perception OR self-concept OR positive affect OR negative affect OR strengths and difficulties OR psychosocial OR social emotional OR psychological OR wellbeing OR child behavio?r checklist OR behavio?ral problem* OR social health OR social problem* OR prosocial OR somatic OR emotion* OR conduct problem* OR hyperactivity OR peer relationship* OR cognitive OR academic achievement OR executive function* OR memory OR inhibition OR attention OR concentrat* OR brain function* OR intelligence OR problem solv* OR perceptual OR life satisfaction OR satisfaction with life |
| 3 | child* OR preschool* OR adolescen* OR teen* OR youth* OR boy* OR girl* OR school-age* OR student* OR p?ediatric or early year* OR elementary school* OR primary school* OR secondary school* OR high school* OR young people OR young person OR toddler* |
| 4 | 1 AND 2 AND 3 |
| 5 | Limit 4 to published 2015-current |
