# Supplementary material for: Association Between 24‐h Movement Behaviors and Mental Health in Children and Adolescents: A Systematic Review and Compositional Data Meta‐Analysis
Source: Scand J Med Sci Sports. 2025 Aug 19;35(8):e70120. doi: 10.1111/sms.70120 (PMC12363385; doi:10.1111/sms.70120)
Supplement: Supplementary file 2 — Appendix B1: supinfo/sms70120‐sup‐0002‐AppendixB1.docx. [file SMS-35-e70120-s004.docx]

**Appendix B**

*Study quality assessment*

**Table B1**

*Study quality assessment for included studies*

| **Study** | **1** | **2** | **3** | **4** | **5** | **6** | **7** | **8** | **9** | **10** | **11** | **12** | **13** | **14** | **15** | **16** | **17** | **18** | **19** | **20** | **21(L)** | **22(L)** | **23(L)** | **OVR** |
| --- | --- | --- | --- | --- | --- | --- | --- | --- | --- | --- | --- | --- | --- | --- | --- | --- | --- | --- | --- | --- | --- | --- | --- | --- |
| Bezerra et al. (2021) | Y | Y | N | Y | Y | N | N | Y | Y | Y | Y | Y | Y | N | Y | Y | Y | Y | Y | Y | NA | NA | NA | 0.75 |
| Bourke et al. (2024) | Y | Y | N | Y | Y | Y | Y | Y | Y | Y | Y | Y | Y | Y | Y | Y | Y | Y | Y | Y | N | Y | Y | 0.87 |
| Carson, Tremblay, Chaput, and Chastin (2016) | Y | Y | N | Y | Y | Y | N | Y | Y | Y | Y | Y | Y | N | Y | Y | Y | Y | Y | Y | NA | NA | NA | 0.80 |
| Chong, Parrish, Cliff, Dumuid, and Okely (2021) | Y | Y | N | Y | Y | N | N | Y | Y | Y | Y | Y | N | N | Y | Y | Y | Y | Y | Y | Y | Y | Y | 0.83 |
| de Faria et al. (2022) | Y | Y | Y | N | N | Y | Y | Y | Y | Y | Y | Y | Y | Y | Y | N | Y | Y | Y | Y | NA | NA | NA | 0.80 |
| Fairclough et al. (2021) | Y | Y | N | Y | Y | Y | N | Y | Y | Y | Y | Y | Y | Y | Y | N | Y | Y | Y | Y | NA | NA | NA | 0.80 |
| Kuzik, Naylor, Spence, and Carson (2020) | Y | Y | N | Y | N | N | N | Y | Y | Y | Y | N | N | N | Y | Y | Y | Y | Y | Y | NA | NA | NA | 0.70 |
| Lau et al. (2024) | Y | Y | N | Y | Y | N | N | Y | Y | Y | Y | Y | N | N | Y | Y | Y | Y | N | Y | NA | NA | NA | 0.75 |
| Li et al. (2024) | Y | Y | N | Y | Y | Y | N | Y | Y | Y | Y | Y | Y | N | Y | Y | Y | Y | Y | Y | NA | NA | NA | 0.80 |
| Lu et al. (2023) | Y | Y | Y | Y | N | N | Y | Y | Y | Y | Y | Y | N | N | Y | Y | Y | Y | Y | Y | NA | NA | NA | 0.85 |
| Lu, Huang, Lou, Li, and Zhou (2024) | Y | Y | N | Y | Y | N | N | Y | Y | Y | Y | Y | N | N | Y | Y | Y | Y | Y | Y | NA | NA | NA | 0.80 |
| Ng et al. (2021) | Y | Y | N | Y | Y | Y | N | Y | Y | Y | Y | Y | N | Y | Y | Y | Y | Y | Y | Y | NA | NA | NA | 0.90 |
| Padmapriya et al. (2024) | Y | Y | N | Y | Y | Y | Y | Y | Y | Y | Y | Y | Y | Y | Y | Y | Y | Y | Y | Y | N | Y | NR | 0.83 |
| Rorem et al. (2024) | Y | Y | N | Y | Y | N | Y | Y | Y | Y | Y | Y | N | Y | Y | Y | Y | Y | Y | Y | NA | NA | NA | 0.90 |
| St. Laurent et al. (2023) | Y | Y | N | Y | N | N | N | Y | Y | Y | Y | Y | N | N | Y | N | Y | Y | Y | Y | NA | NA | NA | 0.70 |
| Tan et al. (2023) | Y | Y | N | Y | Y | Y | Y | Y | Y | Y | Y | Y | Y | Y | Y | Y | N | Y | Y | Y | N | Y | Y | 0.83 |
| Taylor et al. (2023) | Y | Y | N | Y | Y | Y | N | Y | Y | Y | Y | Y | N | Y | Y | Y | Y | Y | Y | Y | N | Y | Y | 0.87 |
| Watson, Dumuid, and Olds (2020) | Y | Y | N | Y | Y | Y | N | Y | Y | Y | Y | Y | N | N | Y | Y | Y | Y | Y | Y | NA | NA | NA | 0.85 |
| Zahran et al. (2024) | Y | Y | N | N | N | N | Y | Y | Y | Y | Y | Y | Y | Y | Y | Y | Y | Y | Y | Y | NA | NA | NA | 0.75 |

Criteria: *1*. Were the aims/objectives of the study clear? *2*. Was the study design appropriate for the stated aim(s)? *3*. Was the sample size justified? *4*. Was the target/reference population clearly defined? (Is it clear who the research was about?) *5*. Was the sample frame taken from an appropriate population base so that it closely represented the target/reference population under investigation? *6.* Was the selection process likely to select subjects/participants that were representative of the target/reference population under investigation? *7*. Were measures undertaken to address and categorise non-responders? *8*. Were the risk factor and outcome variables measured appropriate to the aims of the study? *9*. Were the risk factor and outcome variables measured correctly using instruments/ measurements that had been trialled, piloted or published previously? *10.* Is it clear what was used to determined statistical significance and/or precision estimates? (eg, p values, CIs) *11*. Were the methods (including statistical methods) sufficiently described to enable them to be repeated? *12*. Were the basic data adequately described? *13*. Does the response rate raise concerns about non-response bias? *14*. If appropriate, was information about non-responders described? *15*. Were the results internally consistent? *16*. Were the results for the analyses described in the methods presented? 17*.* Were the authors’ discussions and conclusions justified by the results? *18*. Were the limitations of the study discussed? *19*. Were there any funding sources or conflicts of interest that may affect the authors’ interpretation of the results? *20*. Was ethical approval or consent of participants attained? *21.* Were baseline levels of the outcome controlled for in the analysis? *22.* Was follow-up long enough for one to reasonably expect to see an association between exposure and outcome if it existed? *23.* Was loss to follow-up not related to either the exposure or outcome?

Note. (L) indicated that the criteria only apply to longitudinal studies, Y = yes the study achieved the criteria, N = no the study did not achieve the criteria, NA = not applicable, NR = not reported.

**References**

Bezerra, T. A., Clark, C. C. T., Souza Filho, A. N., Fortes, L. S., Mota, J., Duncan, M. J., & Martins, C. M. L. (2021). 24-hour movement behaviour and executive function in preschoolers: A compositional and isotemporal reallocation analysis. *Eur J Sport Sci, 21*(7), 1064-1072. <https://doi.org/10.1080/17461391.2020.1795274>

Bourke, M., Alsop, T., Peters, R. L., Cassim, R., Wake, M., Tang, M. L. K., & Koplin, J. J. (2024). The cross-sectional and longitudinal association between 24-hour movement behavior compositions with body mass index, waist circumference, and internalizing and externalizing symptoms in 6-year-old children. *Journal of Physical Activity and Health*, 1-13. <https://doi.org/10.1123/jpah.2024-0482>

Carson, V., Tremblay, M. S., Chaput, J.-P., & Chastin, S. F. M. (2016). Associations between sleep duration, sedentary time, physical activity, and health indicators among canadian children and youth using compositional analyses. *Applied Physiology, Nutrition, and Metabolism, 41*(6 (Suppl. 3)), S294-S302. <https://doi.org/10.1139/apnm-2016-0026>

Chong, K. H., Parrish, A. M., Cliff, D. P., Dumuid, D., & Okely, A. D. (2021). Cross-sectional and longitudinal associations between 24-hour movement behaviours, recreational screen use and psychosocial health outcomes in children: A compositional data analysis approach. *Int J Environ Res Public Health, 18*(11). <https://doi.org/10.3390/ijerph18115995>

de Faria, F. R., Barbosa, D., Howe, C. A., Canabrava, K. L. R., Sasaki, J. E., & Dos Santos Amorim, P. R. (2022). Time-use movement behaviors are associated with scores of depression/anxiety among adolescents: A compositional data analysis. *PLOS ONE, 17*(12), e0279401. <https://doi.org/10.1371/journal.pone.0279401>

Fairclough, S. J., Tyler, R., Dainty, J. R., Dumuid, D., Richardson, C., Shepstone, L., & Atkin, A. J. (2021). Cross-sectional associations between 24-hour activity behaviours and mental health indicators in children and adolescents: A compositional data analysis. *J Sports Sci, 39*(14), 1602-1614. <https://doi.org/10.1080/02640414.2021.1890351>

Kuzik, N., Naylor, P. J., Spence, J. C., & Carson, V. (2020). Movement behaviours and physical, cognitive, and social-emotional development in preschool-aged children: Cross-sectional associations using compositional analyses. *PLOS ONE, 15*(8), e0237945. <https://doi.org/10.1371/journal.pone.0237945>

Lau, P. W. C., Song, H., Song, D., Wang, J. J., Zhen, S., Shi, L., & Yu, R. (2024). 24-hour movement behaviors and executive functions in preschoolers: A compositional and isotemporal reallocation analysis. *Child Dev, 95*(2), e110-e121. <https://doi.org/10.1111/cdev.14013>

Li, F., Yin, L., Luo, W., Gao, Z., Ryu, S., Sun, M., . . . Yang, Z. (2024). Isotemporal substitution effect of 24-hour movement behavior on the mental health of chinese preschool children. *Frontiers in Public Health, 12*. <https://doi.org/10.3389/fpubh.2024.1288262>

Lu, B., Huang, Z., Lou, J., Li, R., & Zhou, Y. (2024). Associations between 24-hour activity behaviours and emotional and behavioural problems of left-behind children: A component analysis of data from a cross-sectional study. *BMJ open, 14*(8), e084749. <https://doi.org/10.1136/bmjopen-2024-084749>

Lu, Z., Qu, X., Chang, J., Xu, M., Song, G., Wang, X., . . . Wu, J. (2023). Reallocation of time between preschoolers’ 24-h movement behaviours and executive functions: A compositional data analysis. *Journal of Sports Sciences, 41*(12), 1187-1195. <https://doi.org/10.1080/02640414.2023.2260632>

Ng, E., Wake, M., Olds, T., Lycett, K., Edwards, B., Le, H., & Dumuid, D. (2021). Equivalence curves for healthy lifestyle choices. *Pediatrics, 147*(4). <https://doi.org/10.1542/peds.2020-025395>

Padmapriya, N., Bernard, J. Y., Tan, S. Y. X., Chu, A. H. Y., Goh, C. M. J. L., Tan, S. L., . . . Müller-Riemenschneider, F. (2024). The prospective associations of 24-hour movement behaviors and domain-specific activities with executive function and academic achievement among school-aged children in singapore. *Frontiers in Public Health, 12*. <https://doi.org/10.3389/fpubh.2024.1412634>

Rorem, D., Ezeugwu, V. E., Joly, V. J., Rasmussen, C., Carson, V., Tamana, S. K., . . . Pei, J. (2024). Finding the balance: The influence of movement behaviours on childhood behaviour problems. *Mental Health and Physical Activity, 26*, 100593. <https://doi.org/https://doi.org/10.1016/j.mhpa.2024.100593>

St. Laurent, C. W., Rasmussen, C. L., Holmes, J. F., Cremone-Caira, A., Kurdziel, L. B. F., Desrochers, P. C., & Spencer, R. M. C. (2023). Associations of activity, sedentary, and sleep behaviors with cognitive and social-emotional health in early childhood. *Journal of Activity, Sedentary and Sleep Behaviors, 2*(1), 7. <https://doi.org/10.1186/s44167-023-00016-6>

Tan, S. Y. X., Padmapriya, N., Bernard, J. Y., Toh, J. Y., Wee, H.-L., Tan, K. H., . . . Müller-Riemenschneider, F. (2023). Cross-sectional and prospective associations between children's 24-h time use and their health-related quality of life: A compositional isotemporal substitution approach. *The Lancet Regional Health – Western Pacific, 41*. <https://doi.org/10.1016/j.lanwpc.2023.100918>

Taylor, R. W., Haszard, J. J., Meredith-Jones, K. A., Azeem, A. A., Galland, B. C., Heath, A.-L. M., . . . Healey, D. (2023). Associations between activity, sedentary and sleep behaviours and psychosocial health in young children: A longitudinal compositional time-use study. *Journal of Activity, Sedentary and Sleep Behaviors, 2*(1), 3. <https://doi.org/10.1186/s44167-022-00011-3>

Watson, A., Dumuid, D., & Olds, T. (2020). Associations between 24-hour time use and academic achievement in australian primary school–aged children. *Health Education & Behavior, 47*(6), 905-913. <https://doi.org/10.1177/1090198120952041>

Zahran, S., Cliff, D. P., Antczak, D., Aadland, E., Aadland, K. N., Burley, J., . . . Janssen, I. (2024). Optimal levels of sleep, sedentary behaviour, and physical activity needed to support cognitive function in children of the early years. *BMC Pediatrics, 24*(1), 735. <https://doi.org/10.1186/s12887-024-05186-z>
