# Supplementary material for: Association Between 24‐h Movement Behaviors and Mental Health in Children and Adolescents: A Systematic Review and Compositional Data Meta‐Analysis
Source: Scand J Med Sci Sports. 2025 Aug 19;35(8):e70120. doi: 10.1111/sms.70120 (PMC12363385; doi:10.1111/sms.70120)
Supplement: Supplementary file 3 — Appendix C1: supinfo/sms70120‐sup‐0003‐AppendixC1.docx. [file SMS-35-e70120-s002.docx]

**Appendix C**

*Forrest plots of individual effect sizes based on 30 minute reallocations*

**Figure C1**

*Forest plot of individual effect sizes for spending 30 more minutes sleeping relative to other movement behaviours on social-emotional health*
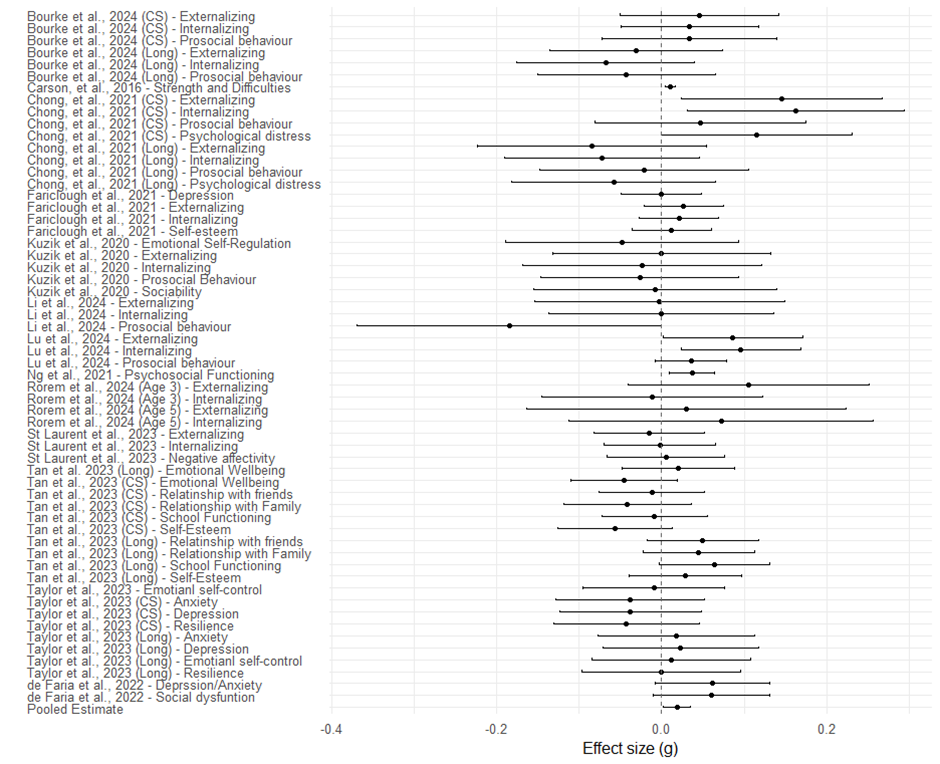


Note. All effect sizes scored so a positive effect size is favourable. CS= cross-sectional, Long = longitudinal.

**Figure C2**

*Forest plot of individual effect sizes for spending 30 more minutes sedentary relative to other movement behaviours on social-emotional health*


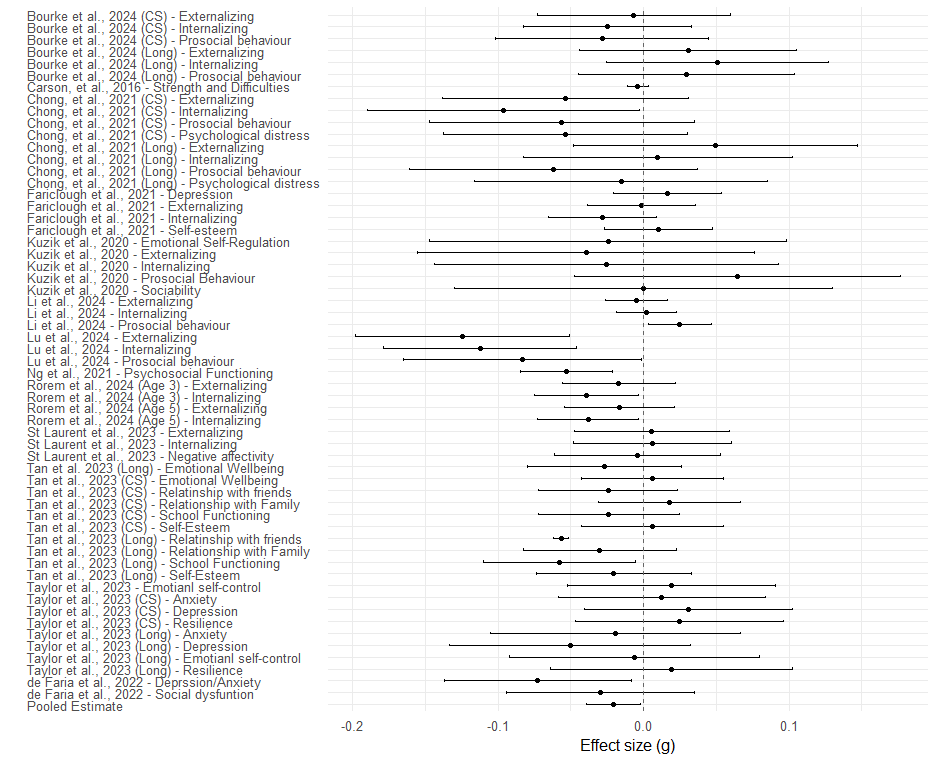


Note. All effect sizes scored so a positive effect size is favourable. CS= cross-sectional, Long = longitudinal.

**Figure C3**

*Forest plot of individual effect sizes for spending 30 more minutes in light intensity physical activity relative to other movement behaviours on social-emotional health*


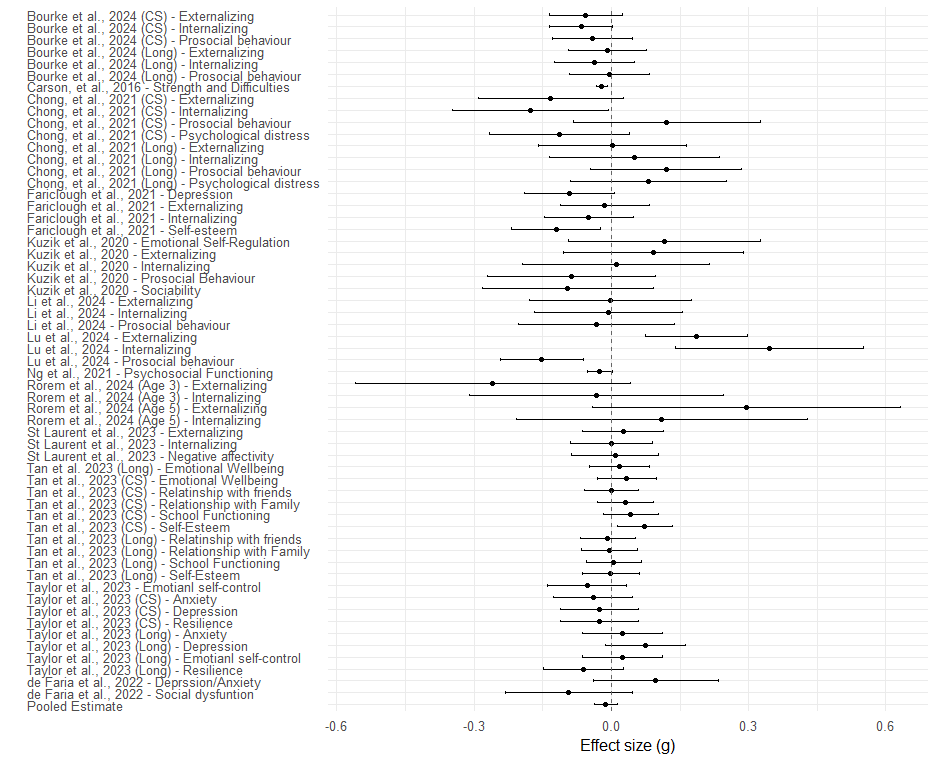


Note. All effect sizes scored so a positive effect size is favourable. CS= cross-sectional, Long = longitudinal.

**Figure C4**

*Forest plot of individual effect sizes for spending 30 more minutes in moderate-to-vigorous physical activity relative to other movement behaviours on social-emotional health*


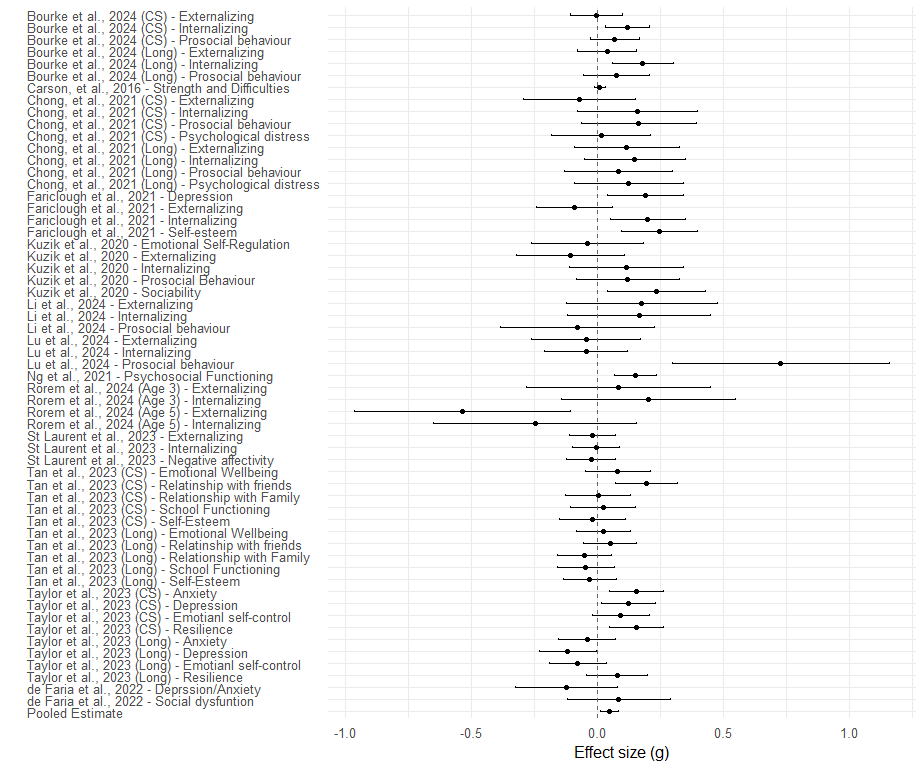


Note. All effect sizes scored so a positive effect size is favourable. CS= cross-sectional, Long = longitudinal.

**Figure C5**

*Forest plot of individual effect sizes for spending 30 more minutes sleeping relative to other movement behaviours on cognitive development*

**
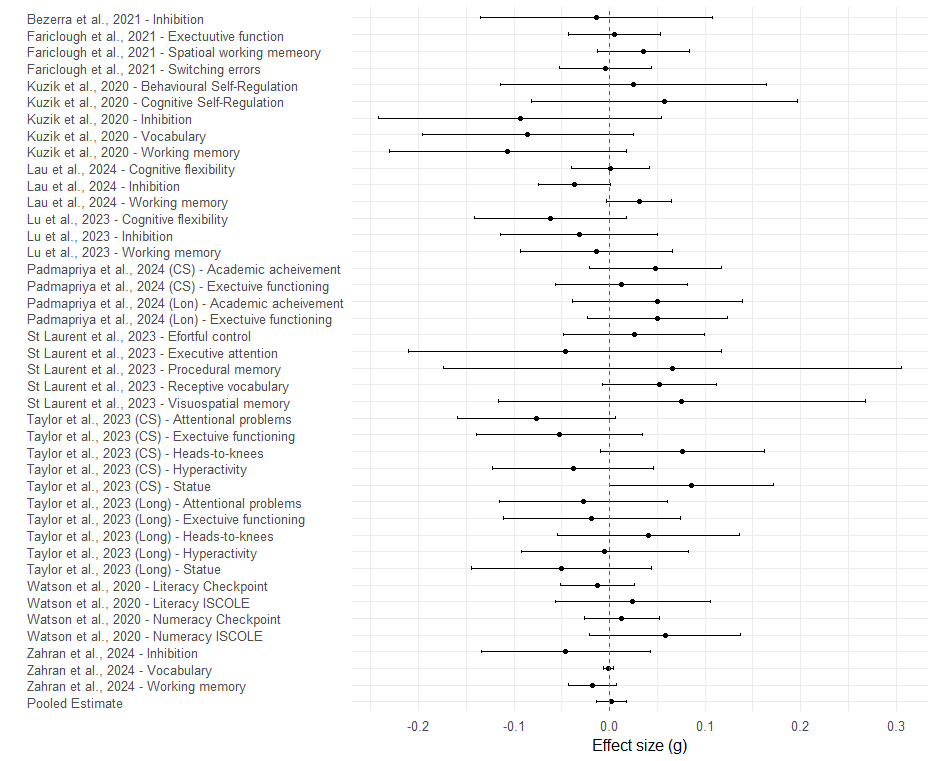
**

Note. All effect sizes scored so a positive effect size is favourable. CS= cross-sectional, Long = longitudinal.

**Figure C6**

*Forest plot of individual effect sizes for spending 30 more minutes sedentary relative to other movement behaviours on cognitive development*

**
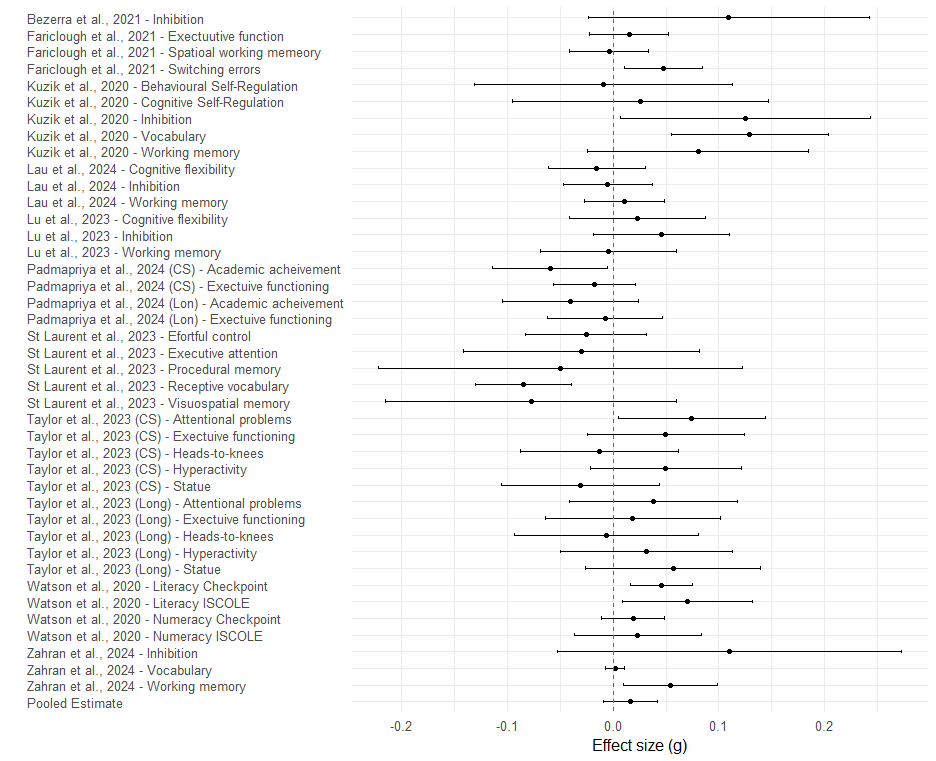
**

Note. All effect sizes scored so a positive effect size is favourable. CS= cross-sectional, Long = longitudinal.

**Figure C7**

*Forest plot of individual effect sizes for spending 30 more minutes in light intensity physical activity relative to other movement behaviours on cognitive development*


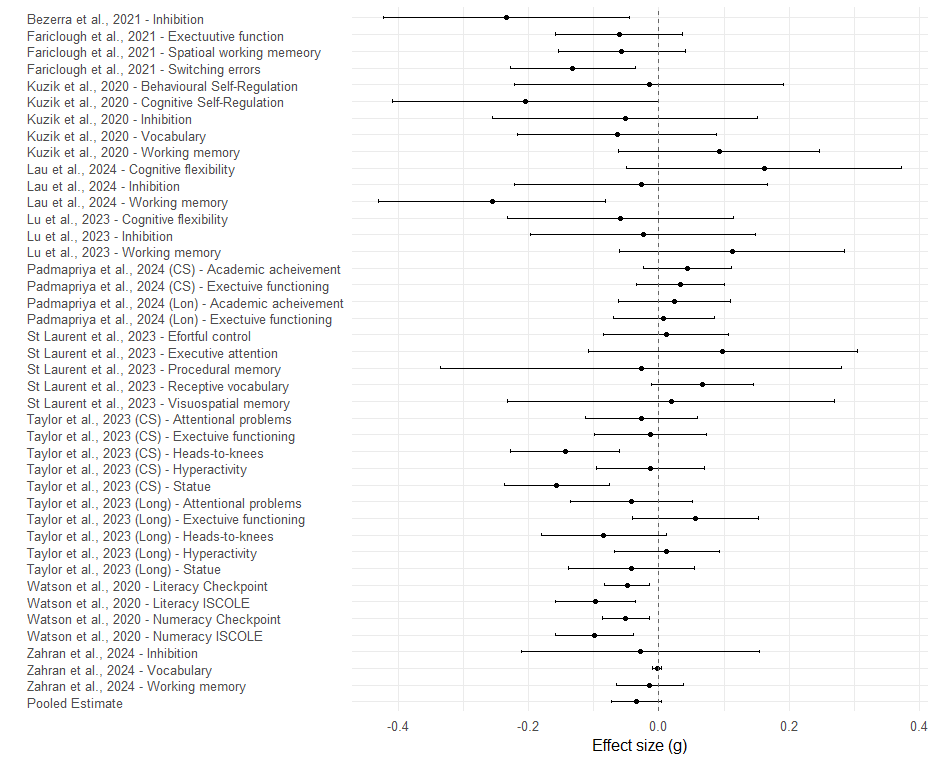


Note. All effect sizes scored so a positive effect size is favourable. CS= cross-sectional, Long = longitudinal.

**Figure C8**

*Forest plot of individual effect sizes for spending 30 more minutes in moderate-to-vigorous physical activity relative to other movement behaviours on cognitive development*
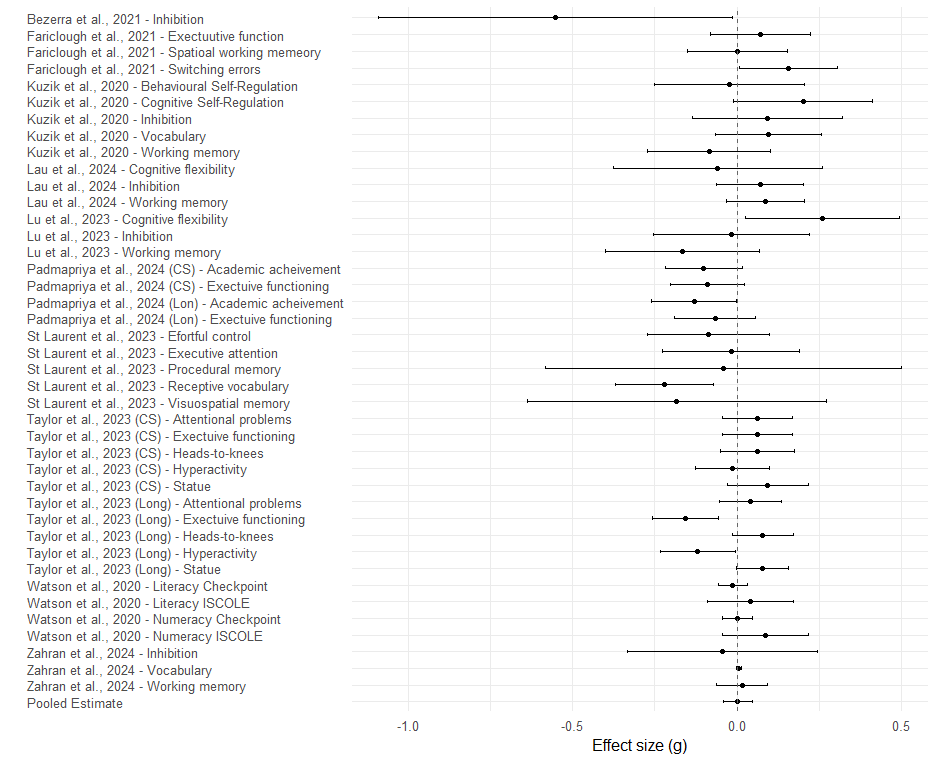


Note. All effect sizes scored so a positive effect size is favourable. CS= cross-sectional, Long = longitudinal.
