# Supplementary figures and images for: Association Between 24‐h Movement Behaviors and Mental Health in Children and Adolescents: A Systematic Review and Compositional Data Meta‐Analysis
Source: Scand J Med Sci Sports. 2025 Aug 19;35(8):e70120. doi: 10.1111/sms.70120 (PMC12363385; doi:10.1111/sms.70120)

**Appendix D**

*Funnel plots of effect sizes and standard errors*


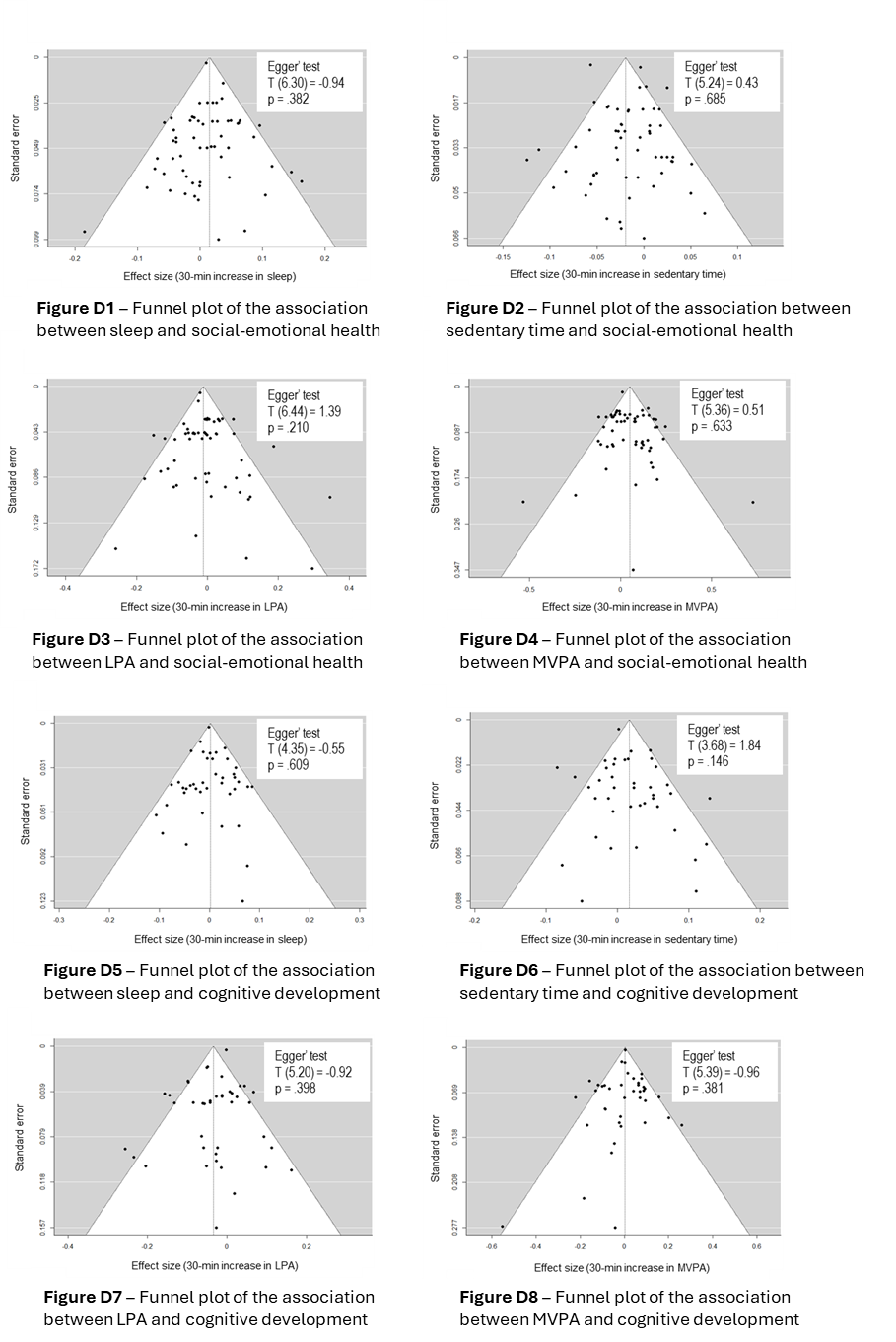

Supplement: Supplementary file 4 — Appendix D1: supinfo/sms70120‐sup‐0004‐AppendixD1.docx. [file SMS-35-e70120-s005.docx]
